# Regulation of mitochondrial cristae organization by Myo19, Miro1 and Miro2, and metaxin 3

**DOI:** 10.1242/jcs.263637

**Published:** 2025-03-06

**Authors:** Samruddhi S. Shembekar, Petra Nikolaus, Ulrike Honnert, Marcus Höring, Aya Attia, Karin Topp, Birgit Lohmann, Gerhard Liebisch, Martin Bähler

**Affiliations:** ^1^ Institute of Integrative Cell Biology and Physiology, University of Münster, 48149 Münster, Germany; ^2^ Institute of Clinical Chemistry and Laboratory Medicine, Regensburg University Hospital, 93053 Regensburg, Germany

**Keywords:** Myosin, Mitochondria, Rho GTPases, Lipid transfer, Cristae organization, ER–mitochondria contact sites

## Abstract

The actin-based motor myosin-19 (Myo19) exerts force on mitochondrial membrane receptors Miro1/2, influencing endoplasmic reticulum (ER)–mitochondria contact sites and mitochondrial cristae structure. The mitochondrial intermembrane bridging (MIB) complex connects the outer and inner mitochondrial membranes at the cristae junction through the mitochondrial contact site and cristae organization system (MICOS). However, the interaction between Myo19, Miro1 and Miro2 (hereafter Miro1/2), and the MIB–MICOS complex in cristae regulation remains unclear. This study investigates the roles of Miro1/2 and metaxin 3 (Mtx3), a MIB complex component, in linking Myo19 to MIB–MICOS. We show that Miro1/2 interact with Myo19 and the MIB complex but not with Mtx3. Their mitochondrial membrane anchors are not essential for MIB interaction or cristae structure. However, Mtx3 is crucial for the connection between MIB–MICOS and the Myo19 and Miro1/2 proteins. Deleting Miro1/2 mimics the effects of Myo19 deficiency on ER–mitochondria contacts and cristae structure, whereas Mtx3 deletion does not. Notably, the loss of Myo19 and Miro1/2 alters mitochondrial lipid composition, reducing cardiolipin and its precursors, suggesting Myo19 and Miro1/2 influence cristae indirectly via lipid transfer at ER–mitochondria contact sites.

## Teaser

**Summary:** Myo19 and Miro1/2 regulate mitochondrial cristae organization via ER–mitochondrial membrane contact mediated lipid transfer, but not via the interaction with the intermembrane bridging complex component Mtx3.

## INTRODUCTION

Mitochondria are indispensable cell organelles due to their role in ATP production, cell death regulation, Ca^+2^ signaling and metabolism. Mitochondria are transported through the mammalian cell with the help of microtubule-based motors. Actin filaments are involved in fission–fusion of mitochondria and serve as tracks for short-range movement. The actin-based motor myosin 19 (Myo19) is associated with mitochondria (Quintero et al., 2009). Mitochondrial outer membrane proteins, GTPases Miro1 and Miro2 (also known as Rhot1 and Rhot2, respectively; hereafter collectively Miro1/2) are the receptors for Myo19 (López–Doménech et al., 2018; Oeding et al., 2018). Notably, absence of Miro1 and Miro2 induces the downregulation of Myo19 and a striking disruption in inner mitochondrial membrane cristae organization (Modi et al., 2019; Shi et al., 2022). Miro proteins have been reported to form a complex with mitochondrial contact site and cristae organization system (MICOS) proteins to maintain the structural organization of cristae (Tsai et al., 2017; Modi et al., 2019). Recently, it has been shown that Myo19 also associates with MICOS proteins and localizes close to crista junctions (Shi et al., 2022). These data establish that Myo19 regulates the inner membrane architecture of mitochondria, raising the question how Myo19 on the outer mitochondrial membrane (OMM) can regulate cristae organization of the inner mitochondrial membrane (IMM).

The mitochondrial intermembrane bridging (MIB) complex is a megadalton supercomplex consisting of the MICOS complex inserted in the inner mitochondrial membrane, the sorting and assembly machinery (SAM) complex inserted in the outer mitochondrial membrane and some additional proteins associated with it on the outer mitochondrial membrane, such as metaxins and DnaJC11 (Xie et al., 2007; Ding et al., 2015; Ott et al., 2015). Interestingly, metaxin 3 (Mtx3) has been suggested to represent a binding partner of Myo19 (Oeding et al., 2018; Bocanegra et al., 2020; Shi et al., 2022; Coscia et al., 2023). The presence of this MIB supercomplex ensures tight regulation of the intermembrane distance between OMM and IMM, which in turn is essential for transport of metabolites and lipids from the OMM to IMM and then to the mitochondrial matrix. Mitochondria can exchange components with other organelles via membrane contact sites. In particular, endoplasmic reticulum mitochondria contact sites (ERMCS) are well-studied contact sites that facilitate the transfer of ions and phospholipids between the two organelles (Kornmann and Walter, 2010; Kojima et al., 2016; Xue et al., 2017). Myo19 and Miro1/2 have been attributed as components of ERMCS in various proteomic screens (Hung et al., 2017; Antonicka et al., 2020; Cho et al., 2020). Furthermore, deletion of Myo19 or Miro1/2 reduced the number of ER–mitochondria membrane contacts (Modi et al., 2019; Coscia et al., 2023; Attia et al., 2024 preprint). Therefore, Myo19 might associate with MIB and ERMCS and thereby influence cristae organization.

In the present work, we investigated the roles of Miro1/2 and Mtx3 in connecting Myo19 to the MIB complex and in controlling cristae organization. To address their roles, we created Miro1/2 double-knockout (Miro DKO) and Mtx3-knockout cells and attempted to rescue them with different constructs. Furthermore, we determined lipid compositions of purified mitochondria.

## RESULTS

### Generation of Miro DKO cells and rescue of the association with the MIB-supercomplex with tail-anchor mutants of Miro1 and Miro2

Myo19 has been shown to contribute to the maintenance of normal cristae architecture (Shi et al., 2022; Attia et al., 2024 preprint). Myo19 binds to the outer mitochondrial membrane proteins Miro1/2, which are required to stabilize Myo19 and protect it from degradation (López-Doménech et al., 2018; Oeding et al., 2018). Deletion of both Miro1 and Miro2 causes a comparable phenotype in cristae organization to that seen upon the loss of Myo19 (Attia et al., 2024 preprint).

**Fig. 1.**
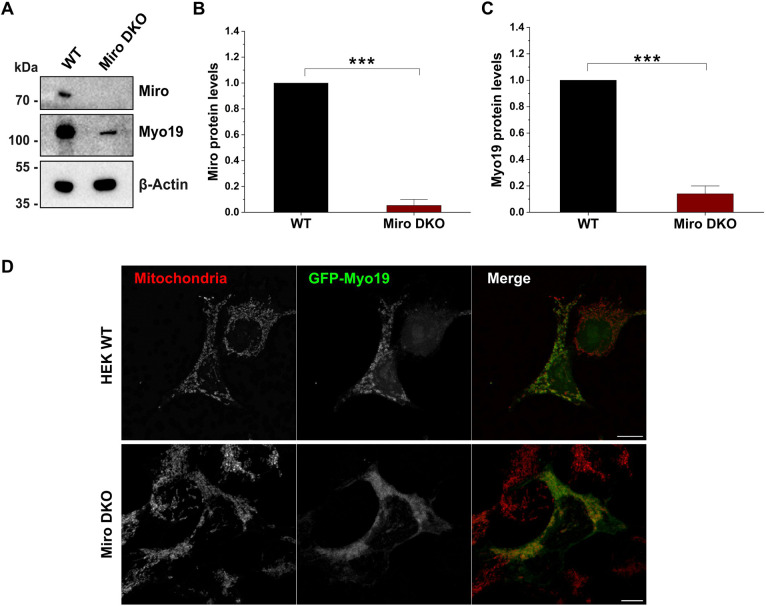
**Generation and characterization of Miro1/2 DKO cells.** (A) Western blot of WT and Miro DKO cell lysates for Miro, Myo19 and β-actin. (B,C) Quantification of Miro and Myo19 protein levels in WT and Miro DKO cells. Protein levels were normalized to β-actin levels. Results are mean±s.e.m. (*n*=3). ***P<0.001 (two-sample unpaired *t*-test). (D) Localization of transiently overexpressed GFP–Myo19 in WT and Miro DKO cells. Mitochondria were stained with Mitotracker Orange. Representative confocal images are shown of GFP–Myo19 in WT and Miro DKO cells. *n*=20–30 cells per experiment (*n*=3 experiments). Scale bars: 10 µm.

The outer mitochondrial membrane receptors Miro1/2 as well as Myo19 have been reported to be associated with the MICOS complex (Tsai et al., 2017; Modi et al., 2019; Shi et al., 2022). Therefore, we wondered whether Myo19 is connected to the MICOS complex through Miro1/2. To address this question, we used a CRISPR-Cas9 approach to delete sequentially Miro1 and Miro2 to create Miro DKO HEK293T cells (Fig. 1A). As shown previously, Miro proteins stabilized Myo19 and the simultaneous loss of both Miro proteins led to the degradation and downregulation of Myo19 (Fig. 1B,C; Lòpez-Domenech et al., 2018; Oeding et al., 2018).

To analyze the localization of Myo19 in wild-type (WT) and Miro DKO cells, we transfected these cells transiently with GFP–Myo19 and stained mitochondria with Mitotracker Orange prior to fixation. Microscopy images revealed that Myo19 in WT cells was exclusively associated with the mitochondria. In contrast, a significant portion of the Myo19 signal was cytosolic in Miro DKO cells, although an association with mitochondria was still discernible (Fig. 1D).

**Fig. 2.**
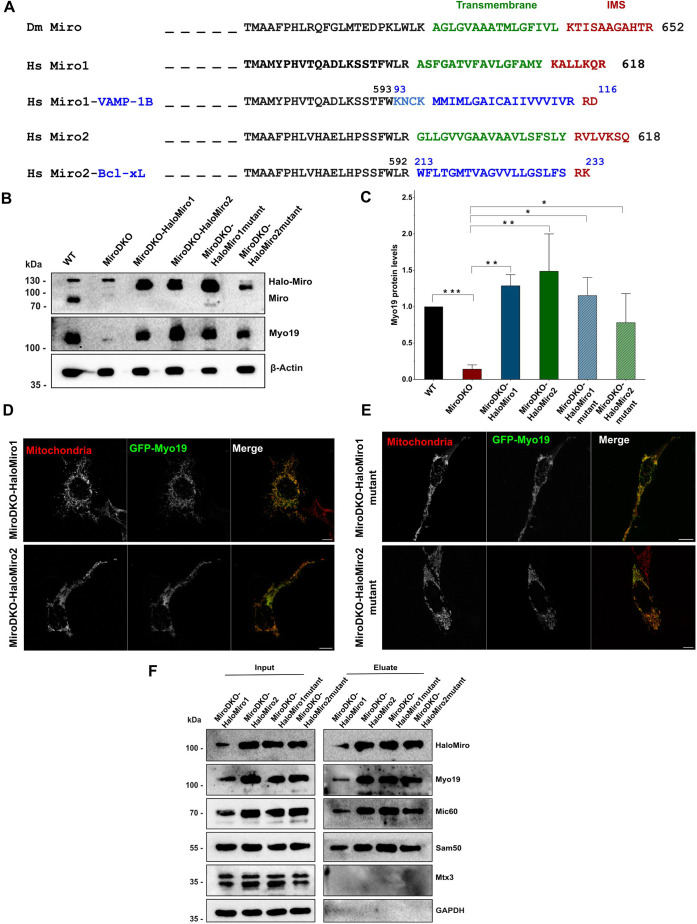
**Membrane anchor mutants of Miro1/2 rescue Miro DKO.** (A) Scheme of Miro1 and Miro2 residues that were exchanged with the corresponding residues of VAMP1B and Bcl-xL, respectively. Residues highlighted in green and blue are part of the transmembrane regions and those in red of the intermembrane space (IMS). Residues in light blue color are derived from VAMP1B and face the cytosol. Dm: *Drosophila melanogaster*, Hs: *Homo sapiens*. (B) Western blot analysis of protein levels of Miro, Halo–Miro, Myo19 and β-actin in WT, Miro DKO and Miro DKO cells expressing different Miro constructs as indicated. (C) Quantification of Myo19 protein levels in the different cell lines as indicated (normalized to β-actin levels). Results are mean±s.e.m. (*n*=3). *P<0.05; **P<0.01; ***P<0.001 (two-sample unpaired *t*-test). (D,E) Confocal images of Miro DKO cells rescued with Halo-tagged Miro constructs (D) and Halo-tagged mutant Miro constructs (E) that were transfected with GFP–Myo19. Mitochondria were stained with Mitotracker Orange. *n*=20–30 cells per experiment (*n*=3 experiments). Scale bars, 10 µm. (F) Miro DKO cells rescued with either Halo–Miro1, Halo–Miro2, Halo–Miro1mutant or Halo–Miro2mutant were lysed. The lysates were subjected to Halo–Trap agarose pull-downs. Western blot analysis of inputs and eluates for Miro, Myo19, Mic60, Sam50, Mtx3 and GAPDH are shown. Images in F are representative of four repeats.

In order to rescue the Miro DKO phenotype, we tagged Miro1 and Miro2 with Halo tag and transfected these constructs individually into Miro DKO cells for stable expression (Fig. 2A). The expression of either Halo–Miro1 or Halo–Miro2 stabilized Myo19 and rescued its expression (Fig. 2B,C) by recruiting it to the mitochondria as verified by the GFP signal after transient transfection of GFP–Myo19 (Fig. 2D). Because upon downregulation of Miro, Myo19 is downregulated concomitantly, we had to take a different approach for analyzing whether Miro links Myo19 to the MICOS complex. Miro is anchored in the outer mitochondrial membrane by a C-terminal transmembrane region (Frederick et al., 2004) with only a very few non-conserved amino acid residues facing the intermembrane space that could possibly associate directly with the MICOS complex (Fig. 2A). To test whether this C-terminal OMM insertion motif of Miro is necessary for the association with MIB or MICOS complex proteins, we replaced it with OMM insertion tail sequences of other C-terminally anchored mitochondrial proteins that are not part of the MIB–MICOS supercomplex. The C-tail region of Miro1 was exchanged with that of VAMP1B (an isoform of VAMP1) and the C-tail region of Miro2 with that of Bcl-xL (an isoform of BCL2L1), respectively (Fig. 2A). These Miro1 and Miro2 tail-mutants were again tagged with Halo tag (Halo–Miro1–VAMP1B tail and Halo–Miro2–Bcl-xL tail) and transfected stably into the Miro DKO cells. To verify the correct mitochondrial localization of Miro1/2 tail mutants, Oregon Green stain was used to label the Halo tags, along with Mitotracker Orange. Halo-tagged tail mutants of Miro1 and Miro2 were colocalized with mitochondria (Fig. S1). Similar to what was seen with WT Miro1/2, the Miro1/2 tail mutants also rescued Myo19 expression (Fig. 2D,E) and Myo19 was localized exclusively to mitochondria in cells expressing the Miro tail mutants (Fig. 2E).

To check the role of Miro1 and Miro2 for the association of Myo19 with the MIB complex, Halo–Trap pulldowns were performed using the cells expressing Halo–Miro1 and Halo–Miro2 in the Miro DKO background. Immunoprecipitation of Halo–Miro1/2 showed that Miro was able to co-precipitate Myo19, Sam50 (also known as SAMM50) and Mic60 (also known as IMMT). Interestingly, Mtx3 was not co-precipitated with either Halo–Miro1 or Halo–Miro2 (Fig. 2F). Halo-Trap pulldown experiments with the Halo-Miro constructs that had replaced their C-terminal transmembrane regions with those of either VAMP-1B or Bcl-xL showed comparable results to pulldowns with WT Miro proteins. Namely, Myo19, Sam50 and Mic60 were co-precipitated, but not Mtx3 (Fig. 2F) indicating that the membrane and intermembrane residues of Miro1/2 are not crucially contributing to the interaction with the MIB–MICOS complex.

### Cristae organization as a function of Miro proteins

**Fig. 3.**
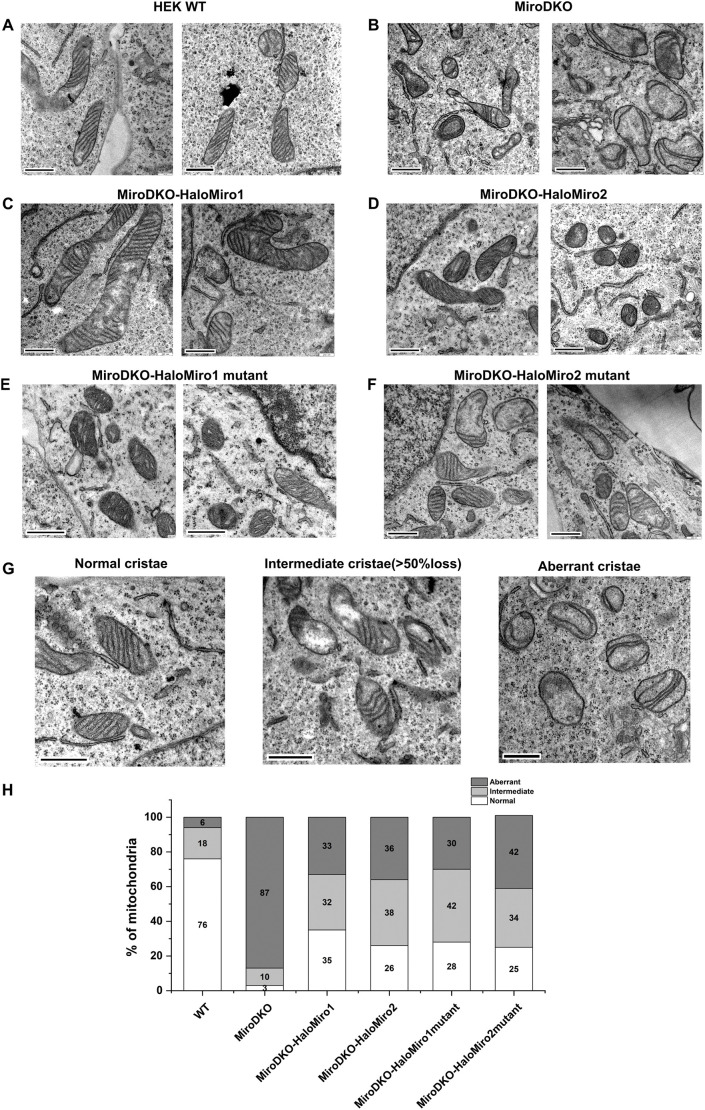
**Ultrastructural analysis of mitochondria in various Miro DKO rescue cells.** Representative TEM images are shown of mitochondria in HEK WT (A), Miro DKO (B), Miro DKO with Halo–Miro1 (MiroDKO-HaloMiro1) (C), Miro DKO with Halo–Miro2 (MiroDKO-HaloMiro2) (D), Miro DKO with Halo–Miro1 mutant (MiroDKO-HaloMiro1 mutant) (E) and Miro DKO with Halo–Miro2 mutant (MiroDKO-HaloMiro2 mutant) cells (F). In G, characteristic examples are shown of mitochondria with normal, intermediate and aberrant cristae organization. (H) Percentages of mitochondria with normal, intermediate and aberrant cristae organization in the indicated cell lines. *n*=200–250 mitochondria for WT and Miro DKO, *n*=150–175 mitochondria for the rescue Miro DKO cell lines. Scale bars: 500 nm.

Previous studies have reported that simultaneous deletion of Miro1 and Miro2 in mouse embryonic fibroblasts disrupts cristae organization (Modi et al., 2019). We have confirmed these results by performing an ultrastructural analysis of our Miro DKO HEK cells (Attia et al., 2024 preprint). We observed a reduction in cristae number and a disruption in cristae organization as compared to mitochondria in WT HEK cells (Fig. 3A,B). Recombinant expression of Miro1 or Miro2 in Miro DKO cells rescued the cristae number and organization in most mitochondria (Fig. 3C,D). Expression of Miro1 and Miro2 mutants that had their membrane anchors replaced rescued cristae organization to the same extent as WT Miro1 and Miro2 (Fig. 3E,F).

For quantitative estimation of the cristae organization in different cell types, we classified the cristae organization into three groups (Fig. 3G): (1) normal (uniformly distributed cristae), (2) intermediate (more than 50% of mitochondrial area does not contain cristae) and (3) aberrant (hardly any cristae). The percentage of aberrant cristae decreased significantly (by 50% as compared to Miro DKO) upon expression of WT or mutant Miro constructs (Fig. 3H). Moreover, the percentage of mitochondria with normal cristae went up from 3% to 30–35% in all of the rescue cell lines (Fig. 3H).

Structural deformations in cristae affect the functionality of mitochondria by hampering ATP production. To see whether the OXPHOS pathway is affected in Miro DKO mitochondria and in the rescues with different Miro constructs, we analyzed oxygen consumption rates (OCRs) with the Agilent Seahorse assay. Consistent with the cristae disorganization, Miro DKO cells demonstrated low OCR as compared to WT cells. Rescue of Miro DKO cells with Halo–Miro1 improved the OCR to levels comparable to that in WT cells. By contrast, the expression of Halo–Miro2 in Miro DKO cells increased OCR only up to 50% of that of WT cells. The membrane anchor mutants of Miro1 and Miro2 also increased OCR considerably (Fig. S2A,B).

### Functional analysis of Mtx3, a member of the MIB complex

**Fig. 4.**
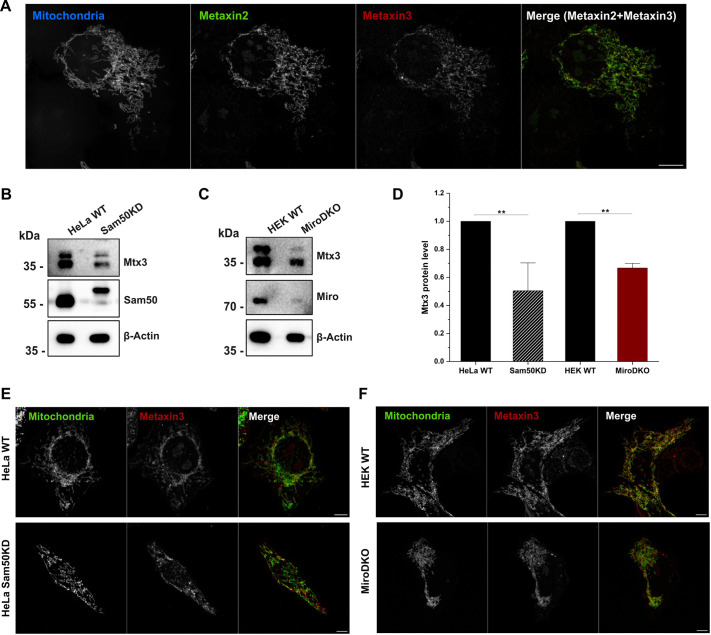
**Analysis of Mtx3 localization and expression.** (A) Representative confocal images showing the colocalization of Mtx2 (green), Mtx3 (red) and mitochondria (blue). Merged image shows colocalization of Mtx2 and Mtx3. Images in A are representative of three experimental repeats. (B) Western blot of HeLa WT and Sam50KD cell lysates probed for Mtx3, Miro and β-actin. (C) Western blot of HEK WT and Miro DKO cell lysates probed for Mtx3, Sam50 and β-actin. (D) Quantification of Mtx3 levels in indicated cell lines (normalized to β-actin). Results are mean±s.e.m. (*n*=3). **P<0.01 (two-sample unpaired *t*-test). (E,F) Representative confocal images of Mtx3–mCherry and MitoBFP (shown in green) in the indicated cell lines, *n*=20–30 cells per experiment (*n*=3 independent experiments). Scale bars: 10 µm.

Metaxin proteins are associated with the outer mitochondrial membrane and were shown to be important for mitochondrial transport in *Caenorhabditis elegans* and human induced pluripotent stem cell (iPSC)-derived neurons (Zhao et al., 2021). A recent study has reported that Mtx3 stabilizes Myo19 on mitochondria (Coscia et al., 2023). Moreover, Mtx3 is believed to be a member of the MIB complex, as shown by complexosome profiling (Huynen et al., 2016), and it represents a potential binding partner of Myo19 (Oeding et al., 2018; Bocanegra et al., 2020; Shi et al., 2022). However, Mtx3 is still poorly characterized. This led us to study Mtx3 in more detail. Confocal microscopy of HEK WT cells co-transfected with metaxin 2 (Mtx2)–GFP and Mtx3–mCherry along with a mitochondrial marker showed clear colocalization of both metaxin proteins with mitochondria (Fig. 4A).

The outer mitochondrial membrane protein SAM50–Mtx2–Mtx3 complex has been reported to be associated with the intermembrane part of the MICOS complex (Huynen et al., 2016; Abudu et al., 2021). Given that Sam50 knockdown (KD) has been shown to downregulate the expression of metaxin 1 and Mtx2 (Abudu et al., 2021), we analyzed the expression of Mtx3 in Sam50 KD cells. Indeed, also Mtx3 was significantly downregulated (Fig. 4B,D). Furthermore, we checked whether the mitochondrial localization of Mtx3 was affected upon knockdown of Sam50. Confocal microscopy analysis of HeLa WT and Sam50 KD cells transfected with Mtx3 and a mitochondria marker showed no significant differences in mitochondrial localization of Mtx3 (Fig. 4E).

Next, we investigated whether Miro proteins not only affect the expression of Myo19, but also the expression of Mtx3. In fact, the expression of Mtx3 was significantly reduced in Miro DKO cells (Fig. 4C,D). However, the association of recombinant Mtx3 with mitochondria was not impaired in Miro DKO cells as shown by confocal fluorescence microscopy (Fig. 4F).

### Mtx3 influences Myo19 expression

**Fig. 5.**
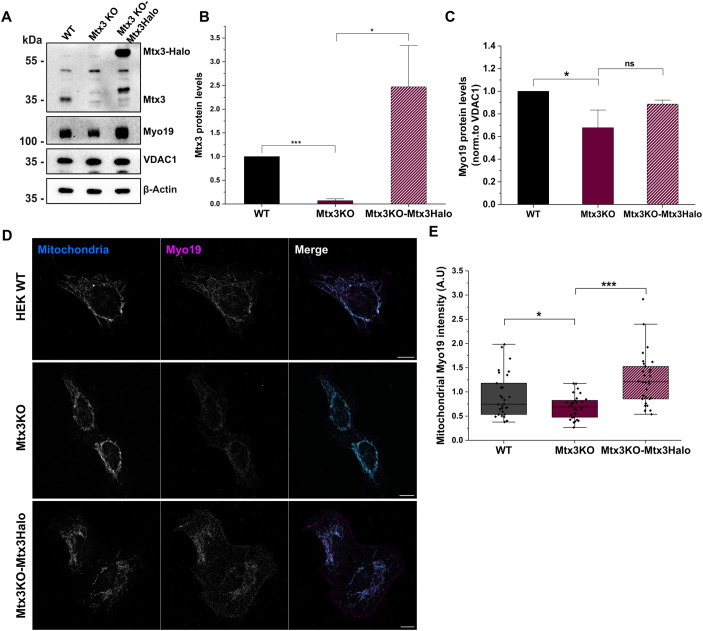
**Myo19 expression and mitochondrial accumulation in Mtx3 KO cells.** (A) Western blot analysis of Mtx3 and Myo19 expression in HEK WT, Mtx3 KO and Mtx3 KO with Mtx3–Halo (Mtx3KO-Mtx3Halo) cells. VDAC1 and β-actin served as controls. (B) Quantification of Mtx3 levels (normalized to β-actin levels) in the indicated cell lines. (C) Quantification of Myo19 levels (normalized to VDAC1 levels) in the indicated cell lines. Results in B and C are mean±s.e.m. (*n*=3). *P<0.05; ***P<0.001; ns, not significant (two-sample unpaired *t*-test). (D) Indirect immunofluorescence analysis of Myo19 (magenta) accumulation on mitochondria stained with Mitotracker Orange (cyan) in HEK WT, Mtx3 KO and Mtx3 KO with Mtx3–Halo cells. Scale bars, 10 µm. (E) Mitochondrial Myo19 intensity was determined applying a mitochondrial mask and is plotted for HEK WT, Mtx3 KO and Mtx3 KO with Mtx3–Halo cells. The box represents the 25–75th percentiles, and the median is indicated. The whiskers show the 10th to 90th percentiles. *P<0.05; ***P<0.001 (one-way ANOVA analysis).

Coscia et al. (2023) has reported recently that Mtx3 stabilizes Myo19 on mitochondria. They had used siRNA-mediated knockdown of Mtx3 to study its impact on Myo19 expression and mitochondrial localization. To extend this study, we engineered a Mtx3-knockout HEK293T cell line and studied Myo19 expression and localization in this cell line. Moreover, we expressed recombinant Mtx3 tagged C-terminally with Halo tag in the Mtx3 KO cells (Mtx3KO-Mtx3Halo) to rescue specifically changes induced by the lack of Mtx3 (Fig. 5A,B). Western blot analysis of cell lysates showed that Myo19 expression was indeed downregulated in Mtx3KO cells and upregulated upon overexpression of Mtx3–Halo (Fig. 5A,C).

Mitochondrial accumulation of Myo19 as a function of Mtx3 expression was checked by immunofluorescence. The mitochondrial intensity of Myo19 was reduced in Mtx3KO cells as compared to WT cells. Conversely, overexpression of Mtx3–Halo in Mtx3KO cells caused a significant increase in mitochondrial Myo19 intensity (Fig. 5D,E).

### Ultrastructural analysis of cristae morphology and number of ERMCS in Mtx3 KO cells

**Fig. 6.**
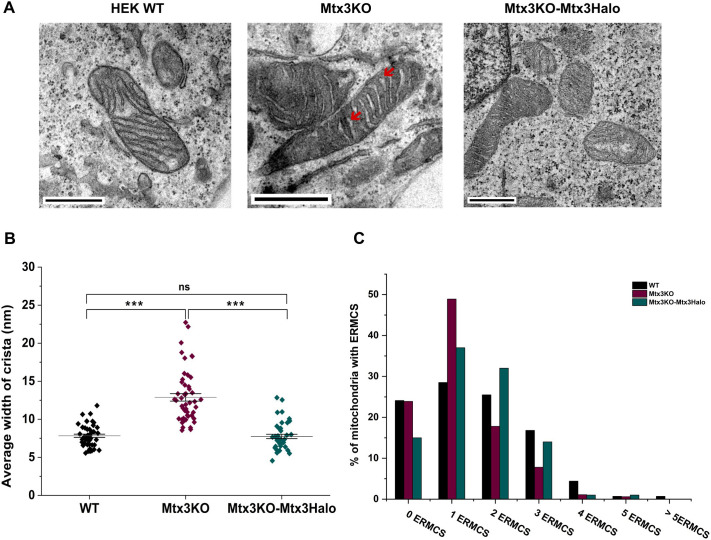
**Mtx3 KO mitochondria show wider cristae and fewer multiple ER contacts.** (A) Representative TEM images of mitochondria in HEK WT, Mtx3 KO and Mtx3 KO with Mtx3–Halo (Mtx3KO-Mtx3Halo) cells. Red arrows point to the enlarged cristae in Mtx3KO mitochondria. Scale bars: 500 nm. (B) Width of each crista per mitochondrium was measured for WT, Mtx3 KO and Mtx3 KO with Mtx3–Halo cells. Average width was calculated and plotted. Results are mean±s.e.m. (*n*=30–40 mitochondria per cell type). ***P<0.001; ns, not significant (one-way ANOVA with Tukey–Kramer post-hoc test). (C) Mitochondria in WT, Mtx3 KO and Mtx3 KO with Mtx3–Halo cells were classified based on the number of contacts with ER (ERMCS). Distribution of mitochondria with given number of ERMCS is shown for *n*=130–150 mitochondria per cell type.

It has been shown that the MIB complex and Myo19 regulate cristae number and morphology (Rampelt et al., 2016; Shi et al., 2022; Attia et al., 2024 preprint). Mtx3 is a member of the MIB complex (Huynen et al., 2016) and it also interacts with Myo19. Therefore, Mtx3 might also influence cristae organization. To verify this, we investigated a potential role of Mtx3 in cristae structure by analyzing the ultrastructure of the mitochondria in WT, Mtx3 KO and Mtx3KO-Mtx3Halo cells. We noticed that the cristae of Mtx3 KO mitochondria (77% of total analyzed mitochondria, Fig. S3C) were significantly wider in comparison to WT mitochondria (Fig. 6A). Measurements of the mean width of cristae per mitochondrium revealed that they were almost twice as wide as those in WT mitochondria. Rescue of Mtx3 KO cells with stable expression of Mtx3–Halo reduced the width of cristae to WT level (Fig. 6B) in ∼75% of the total mitochondria (Fig. S3C). However, we would like to emphasize that the Mtx3 KO cristae phenotype did not reflect the phenotypes observed upon deletion of Myo19 and Miro1/2.

Myo19 has been shown to control the number of ERMCS (Coscia et al., 2023; Attia et al., 2024 preprint). As Mtx3 is located in close proximity to Myo19 on mitochondria, its deletion might also influence the number of ERMCS. Therefore, we analyzed the abundance of mitochondria with or without ERMCS in WT, Mtx3 KO and Mtx3KO-Mtx3Halo cells. Mtx3 KO mitochondria showed more frequently a single ER contact site than WT, but less multiple contact sites (Fig. 6C). Expression of Mtx3–Halo reduced the number of mitochondria with a single contact and increased the number with multiple contacts, confirming that Mtx3 does influence multiplicity of ERMCS (Fig. 6C). One of the possible reasons for differences in ERMCS numbers could be corresponding differences in surface area of the mitochondria. However, we found no significant difference in the area between WT and Mtx3 KO mitochondria (Fig. S3D). Overall Mtx3 might regulate a particular type of mitochondrial ER contact, but again Mtx3 did not regulate the number of ERMCS to the same extent as do Myo19 and Miro1/2 (Attia et al., 2024 preprint; Coscia et al., 2023; Modi et al., 2019).

### Mtx3 regulates the association of Myo19 with the MIB complex

**Fig. 7.**
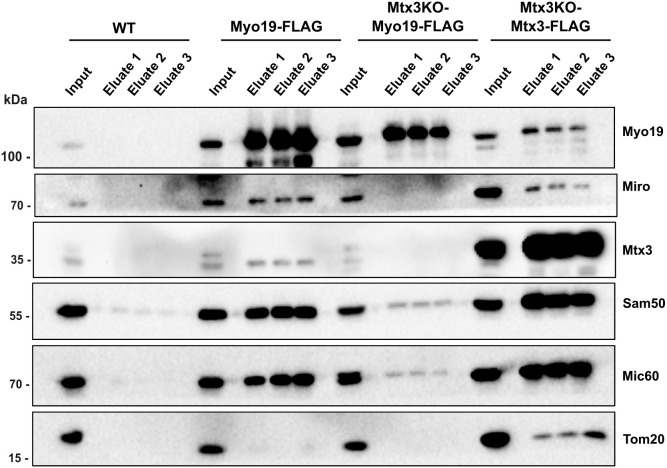
**Association of Myo19 with the MIB supercomplex in the presence and absence of Mtx3.** Myo19–FLAG and Mtx3–FLAG, respectively, were stably expressed in indicated cells and affinity purified with anti-FLAG agarose. HeLa WT cells served as control. Cell lysates and proteins eluted with FLAG-peptide were separated on SDS-PAGE and immunoblotted with the indicated antibodies. Blot shown is representative of three experimental repeats.

Considering the effects of Mtx3 on Myo19 expression, we speculated that it might stabilize the Myo19–Miro interaction on the outer mitochondrial membrane. When we affinity purified Myo19–FLAG stably transfected in HeLa cells, Miro1/2 and Mtx3 were co-purified along with MIB components Sam50 and Mic60 (Fig. 7). Affinity purification by anti-FLAG agarose of Mtx3–FLAG stably transfected into Mtx3 KO cells revealed that Mtx3 was forming a complex with MIB members Sam50 and Mic60 (Fig. 7). However, in the Mtx3–FLAG purifications we also detected Myo19 and small amounts of Miro1/2 (Fig. 7). Next, we asked whether Mtx3 is instrumental for the Myo19–MIB interaction. To this end, we created Mtx3 KO cells stably expressing Myo19–FLAG (Mtx3KO-Myo19-FLAG). Purifications of Myo19 with anti-FLAG agarose in the absence of Mtx3 showed a striking reduction in the amounts of co-purified Sam50 and Mic60 as compared to purifications in the presence of Mtx3 (Fig. 7). Unexpectedly, in the absence of Mtx3, no detectable Miro1/2 was co-purified with Myo19, although Myo19 did interact with Miro1/2 directly (Fig. 7). Altogether, these results suggest that Mtx3 provides an important link between Myo19 and the MIB–MICOS complex, although Miro together with Myo19 appears to be able to associate with the MIB complex independently of Mtx3 (see Fig. 2).

**Fig. 8.**
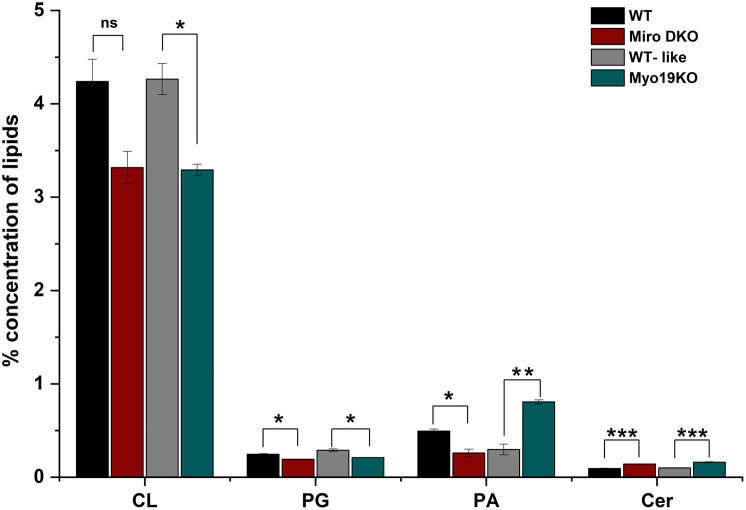
**Lipidomic analysis of WT, Miro DKO and Myo19 KO mitochondria.** (A) Percentage levels of lipids from WT, Miro DKO, WT-like (CRISPR-treated control for Myo19 KO) and Myo19 KO mitochondria plotted as bar graph (mean±s.e.m. *n*=3 independent mitochondria purifications). CL, cardiolipin; PG, phosphatidylglycerol; PA, phosphatidic acid; Cer, ceramide.; *P<0.05; **P<0.01; ***P<0.001; ns, not significant (*t*-test, two-tailed distribution, heteroscedastic).

The cristae organization and the abundance of mitochondria–ER membrane contacts in Mtx3 KO cells differed from those observed in Myo19 KO and Miro1/2 DKO cells. Therefore, we wondered whether the reduced numbers of ERMCS in Myo19 KO and Miro1/2 DKO cells, rather than an impaired interaction with the MIB complex could be responsible for the observed alterations in cristae organization. The reduced numbers of ERMCS could give rise to an altered mitochondrial lipid composition, which in turn would affect cristae organization. Therefore, we analyzed the lipid compositions of purified mitochondria from WT, Myo19 KO and Miro1/2 DKO cells (Fig. 8).

Phosphatidylserine (PS) and phosphatidylethanolamine (PE) are important lipids shuttled from the ER. Deletion of Miro1/2 did not significantly affect the abundance of PS in mitochondria but altered that of PE. Whereas the absence of Myo19 contributed to significant changes in the levels of both PS and PE (Fig. S4). Cardiolipin (CL) is a characteristic lipid of mitochondria found mostly in the inner membrane. It is essential for the maintenance of membrane curvature and for proper localization of ATP synthase units on cristae rims (Schlame and Ren, 2009; Venkatraman et al., 2023). CL synthesis mainly occurs at the IMM from the precursors phosphatidic acid (PA) and phosphatidylglycerol (PG). In the process, mitochondria obtain their PA mainly through transfer from the ER (Flis and Daum, 2013). We found a significant reduction in the levels of CL and PG in Miro DKO mitochondria (Fig. 8). As Miro1/2 deletion strongly downregulates Myo19 expression and reduces the number of ERMCS, mitochondrial CL and PG levels were additionally analyzed for Myo19 KO mitochondria. There was a similar reduction in these lipids as mitochondria from Miro DKO cells (Fig. 8). Increased levels of ceramide are known to negatively affect mitochondrial dynamics and metabolism (Ding et al., 2024). In mitochondria from both Miro DKO and Myo19 KO cells increased amounts of ceramide were detected in purified mitochondria (Fig. 8).

## DISCUSSION

We show here that Myo19 is associated with the MIB–MICOS supercomplex and thereby transmits its pulling force from the OMM to the IMM. Affinity-purified Myo19 is isolated with its OMM receptors Miro1/2, the MIB components Sam50, Mtx3 and the MICOS component Mic60. These results agree well with previous reports that have demonstrated an association of Myo19 and Miro with the MIB–MICOS supercomplex (Modi et al., 2019; Shi et al., 2022). Deletion of Myo19 as well as Miro1/2 results in a clear reduction and alteration in cristae junctions (Attia et al., 2024 preprint; Modi et al., 2019; Shi et al., 2022), suggesting that cristae junction formation might be regulated by actin-based force that is sensed by the MICOS complex. However, deletion of Mtx3, which significantly contributes to the association of Myo19 with the MIB–MICOS supercomplex, did not show a comparable cristae organization phenotype to that seen on the deletion of either Myo19 or Miro1/2. It is currently not known how Miro1/2 and to some extent Myo19 are associated with the MICOS complex. In Miro1/2 pulldowns, Myo19 and components of the MIB/MICOS supercomplex were also co-purified, but interestingly not Mtx3. We could exclude that the membrane anchors of Miro1/2 mediate directly or indirectly the association of Miro1/2 with the MICOS complex. Membrane anchor replacement, including the few residues protruding into the IMS, did not abolish the association of Miro1/2 with the MIB–MICOS supercomplex. Furthermore, this did also not affect Myo19 stability and, more importantly, cristae architecture. This indicates that the cytosolic portion of Miro1/2 accounts for the association with the MIB–MICOS complex and cristae organization. A natural candidate for mediating the association of Miro1/2 with the MIB–MICOS complex could be Mtx3, which is part of the MIB complex (Huynen et al., 2016) and connects Myo19 to Sam50. Mtx3 has been identified in proximity labelling screens with a Myo19 tail fragment (Bocanegra et al., 2020; Oeding et al., 2018). However, Miro1/2 pulldowns did not co-precipitate Mtx3, challenging the idea that Mtx3 is the adaptor that links Miro1/2 via Myo19 to Sam50. Therefore, further work will be needed to identify the adaptor between the Miro1/2 cytosolic region and the MIB–MICOS supercomplex. However, Mtx3 cannot be completely excluded from this role. Mtx3 is the least-studied member of the metaxin family, which additionally contains Mtx1 and Mtx2. To study the role of Mtx3 in more detail, we created a Mtx3 KO cell line and a progeny that expressed recombinant Mtx3–FLAG. The loss of Mtx3 reduced somewhat the level of Myo19. However, the reduction of Myo19 in the absence of Mtx3 was much less strong than in the absence of Miro1/2. The reduction in Myo19 protein level would be compatible with the idea that Mtx3 supports the interaction of Myo19 with Miro1/2. This explanation is further supported by our finding that Miro1/2 were not co-purified with Myo19 when Mtx3 was absent. However, Mtx3 was not detected in pulldowns of Miro1/2, which argues against this explanation. Furthermore, Mtx3 might not be the sole intermediary for the association of Myo19 and Miro1/2 with the MIB–MICOS supercomplex, given that the MIB–MICOS components Sam50 and Mic60 were co-purified to a small extent with Myo19 even in the absence of Mtx3. In addition to the metaxin–Sam50 complex, several other outer mitochondrial membrane proteins have been suggested to interact with the MICOS complex, including the TOM complex (von der Malsburg et al., 2011; Michaud et al., 2016), DNAJC11 (Xie et al., 2007) and SLC25A46 (Harner et al., 2011). It remains to be seen whether any of these proteins contribute to the association of Myo19 and Miro1/2 with the MICOS complex.

In Mtx3 KO cells, the mitochondria changed from an orthodox state, with the narrow cristae and an extended matrix seen in WT and Mtx3KO-Mtx3Halo cells, to a condensed state with expanded cristae volume and a decreased matrix volume (Hackenbrock, 1966; Mannella, 2006). This differs from the clearly altered cristae organization in mitochondria from Myo19 KO and Miro1/2 DKO cells (Attia et al., 2024 preprint; Modi et al., 2019; Shi et al., 2022).

The altered cristae organization in mitochondria of Myo19 KO and Miro1/2 DKO cells could be an indirect consequence of the reduced numbers of ERMCS that were observed in these two cell lines (Modi et al., 2019; Attia et al., 2024 preprint; Coscia et al., 2023). ERMCS are known to mediate lipid transfer between the two organelles (Kornmann et al., 2009; Alva and Lupas, 2016; Kawano et al., 2018). In fact, our analysis of the total lipid composition of mitochondria purified from WT, Myo19 KO and Miro1/2 DKO cells revealed some alterations. In comparison to what was seen in WT mitochondria, there were reduced levels of cardiolipin and a precursor of it. Cardiolipin has been shown to be a crucial determinant of cristae morphology (Jiang et al., 2000; Zhong et al., 2004; Friedman et al., 2015; Rampelt et al., 2018). Therefore, Myo19 and Miro1/2 might regulate cristae organization by regulating the formation of particular ERMCS.

## MATERIALS AND METHODS

### Plasmids

The plasmid pX330-Miro1ex7 encoding for CRISPR-Cas9 mediated knockout of human Miro1 was described by Kanfer et al. (2015) and a gift from Prof. Benoit Kornmann (University of Oxford, UK). A human Miro2 target specific CRISPR-Cas9 knockout plasmid was purchased from Santa Cruz Biotechnology (sc-406496).

Plasmids GFP-Myo19, Mito-mTagBFP2 and pIREShyg-Halo have been described in previous work (Oeding et al., 2018; Majstrowicz et al., 2021).

To prepare pIRES-Myo19-FLAG, a fragment coding for the N-terminal part of Myo19 was isolated from plasmid GFP–Myo19 with BglII and SmaI restriction. The fragment coding for the remaining C-terminal part of Myo19 fused to the FLAG tag was amplified by PCR using Myo19 cDNA as a template, resulting in a PCR product flanked by the restriction sites for SmaI and NheI. A triple ligation of pIREShyg-Linker (digested with BamHI and NheI) and the two fragments resulted in the plasmid pIREShyg-Myo19-FLAG.

Full-length human Miro1 cDNA, previously described in (Oeding et al., 2018), was cut with BglII/XbaI and cloned into vector Halo-pIREShyg via BamHI/NheI, resulting in the plasmid Halo-Miro1-pIREShyg (encoding for Halo-Miro1). Similarly, a plasmid encoding for Halo–Miro2 was prepared using human full-length Miro2 cDNA from the plasmid pRK5-myc-Miro2 (Addgene plasmid #47891) (Fransson et al., 2003). The plasmid mCherry-Miro1 was constructed by inserting Miro-1 into the XhoI and SacII sites of pmCherry-C1. To exchange the DNA sequence coding for the C-terminal tail sequence of Miro1 with that coding for amino acids (aa) 93–116 of VAMP1B, two pairs of primers were annealed, phosphorylated and cloned with Eco72I and Cfr42I by a triple ligation into the plasmid mCherry–Miro1 (now encoding for aa 1–593 of Miro1) to obtain plasmid mCherry-Miro1-VAMP1B tail. Subsequently, the sequence Miro1–VAMP1B tail was excised with BglII and XbaI and cloned into the vector Halo-pIREShyg-linker (cut with BamHI and NheI) to obtain the plasmid Halo-Miro1-VAMP1B tail.

The construct coding for mCherry–Miro2 aa 1–592 and ending with a Bcl-xL transmembrane sequence was prepared in two steps. First, a plasmid containing Miro2 (encoding for aa 1–592), the first five amino acids of the Bcl-xL transmembrane sequence (aa 213–217) and a BshTI restriction site (silent mutation) was constructed with the help of PCR by using pRK5-myc-Miro2 (Addgene #47891) as a template. The PCR product was then cloned with Eco72I and SalI into mCherry-Miro2 aa 1–588 2×FYVE (Oeding et al., 2018). In the second step, the C-terminal part of Bcl-xL (aa 213–233) was created by annealing of two primers. This fragment was then cloned with BshTI and SalI into the construct described above. Halo–Miro2 (aa 1–592)–Bcl-xL was constructed by ligating Miro2 (aa 1–592)–Bcl-xL (cut with BglII and XbaI) into the vector Halo-pIRES hyg Linker (cut with BamHI and NheI).

Mtx2 cDNA was PCR amplified from pDEST15-MTX2 and cloned into pEGFP-N1 (Clontech) with EcoRI and BamHI. pDEST15-MTX2 was a gift from Prof. Terje Johansen (University of Tromsø, Norway). To create pMetaxin 3-mCherry, the full-length cDNA of human Mtx3 was amplified from Mtx3 clone BC160186 (Bio Cat, Troy, USA) by PCR and subcloned into vector pmCherry-N1 (Clontech) using restriction sites XhoI and BamHI.

The plasmid Metaxin 3-Halo-pIREShyg was constructed by restriction of the plasmid Myo19–Halo described previously (Oeding et al., 2018) with BshTI and NheI followed by insertion of the Mtx3 sequence derived from pMetaxin 3-mCherry. Full-length cDNA of human Mtx3 was amplified from Mtx3 clone BC160186 (Bio Cat, Troy, USA) with primer encoding for FLAG tag and NheI and Eco32I restriction sites. This PCR product was then cloned into pIRES hyg-linker (digested with NheI and Eco32I) to prepare pIRES-Mtx3-FLAG.

### Antibodies

The following antibodies were used for western blotting (WB) and immunofluorescence (IF): anti-Myo19 (Abcam ab174286, 1:1000 for WB, 1:250 for IF), anti-Mic60 (Proteintech 10179-1-AP, 1:1000), anti-Sam50 (Proteintech 28679-1-AP, 1:1000), anti-VDAC1 (Abcam ab14734; 1:1000), anti-Miro1/2 (Novus Biologicals NBP1-59021, 1:1000), anti-GAPDH (Proteintech 60004-1-Ig, 1:5000), anti-β-actin (Sigma-Aldrich AC-15/A1978, 1:2000), anti-Tom20 (Santa Cruz Biotechnology sc-17764, 1:500) and anti-Mtx2 (Santa Cruz Biotechnology sc-514231, 1:500) antibodies. Polyclonal anti-Mtx3 antibody was raised by immunizing a rabbit with recombinant Mtx3 fused with a 6×His-tag, expressed in *Escherichia coli* and affinity purified by Talon agarose followed by extraction from SDS-PAGE. Gel extraction and immunization was performed by Davids Biotechnologie (Regensburg, Germany). Rabbit serum after immunization with recombinant Mtx3 was further purified by affinity chromatography.

### Cell culture, generation of knockout and stable cell lines

HEK293T cells (ECACC) and HeLa cells (ATCC) were cultured using DMEM (P04-03550, PAN Biotech) supplemented with 10% fetal bovine serum (Biochrom) along with 100 U/ml penicillin and 100 µg/ml streptomycin (P06-07100, PAN Biotech) (called complete DMEM below). For selection and maintenance of stable cell clones, 100 µg/ml hygromycin (1358GR005, BioFroxx) was additionally added to the cell culture medium. Polyethyleneimine (PEI)-mediated transfection of DNA was carried out in case of transient transfection for pulldown assays. Otherwise, Lipofectamine® LTX Plus Reagent (15338100, Invitrogen) was used for transfections following the protocol provided by the manufacturer.

To create Miro1/2 DKO cells, HEK 293T WT cells were first transfected with pX330-Miro1ex7 (a gift from the Kornmann laboratory, Department of Biochemistry, University of Oxford, UK). The resulting clones after FACS sorting of transfected cells were grown and subsequently checked for Miro1 KO by PCR sequencing and western blotting. The Miro2 CRISPR/Cas9 KO plasmid was obtained from Santa Cruz Biotechnology (sc-431979). Miro1 KO cells were subsequently transfected with this Miro2 KO plasmid to obtain Miro DKO cells. The same procedure as described for obtaining Miro1 KO clones was applied to isolate Miro DKO cell clones. HEK 293T Mtx3 knockout cells were custom-made by Ubigene Biosciences (Ubigene Biosciences Co., Ltd., Austin, USA). Sam50 KD cells were a kind gift from Prof. Terje Johansen (University of Tromsø, Norway).

Rescue of knockout cell lines was performed by stable transfection of cells (100,000 cells in a 6-well plate) with plasmids coding for the appropriate rescue construct. The plasmid was first linearized with a suitable restriction enzyme and then transfected using Lipofectamine^®^ LTX Plus Reagent. Transfected cells were trypsinized after 48 h and then plated in 15-cm culture dishes as 60%, 30% and 10% of the total cells, in DMEM supplemented with 100 µg/ml hygromycin. Cell colonies were selected for 14 days, and surviving clones were later analyzed by western blotting for expression of the recombinant rescue construct.

### Preparation of cell lysates and western blots

Cells were seeded in 6-well dishes and harvested in cold PBS at 70–80% confluency. Cell pellets were washed with cold PBS and then resuspended with NP-40 lysis buffer (50 mM Tris-HCl, pH 7.4, 10% glycerol, 100 mM NaCl, 2 mM MgCl_2_, 1% NP-40 and freshly added 1 mM DTT, 10 µg/ml aprotinin (A162, Roth), 10 µg/ml leupeptin (CN33, Roth) and 10 µg/ml Pefabloc (A154, Roth) to lyse the cells. After brief incubation on ice, an aliquot was diluted for further protein quantification and the rest of the lysate was boiled after adding 5× SDS-Laemmli buffer. Bradford reagent was used to quantify protein content of each sample using BSA as a standard. 10–50 µg of protein was resolved on SDS-PAGE gels and then blotted on PVDF membrane. Membranes were blocked with 5% non-fat milk in Tris-buffered saline with 0.1% Tween-20 (TBST) and then primary antibodies (diluted in blocking buffer) were added overnight at 4°C. After three washes with TBST, secondary antibodies were added (1:5000 diluted in blocking buffer) for 1 h at room temperature (RT). Membranes were again washed three times for 5 min each, with TBST and then Super Signal West Pico Substrate was used to detect chemiluminescent signal with a ChemiDoc MP Imaging System (Bio-Rad). Stripping of PVDF membranes was carried out whenever necessary. Blots were incubated in stripping buffer (50 mM Tris-HCl pH 6.8, 2% SDS and 100 mM β-mercaptoethanol) for 20 min followed by 3× washing with TBST. The unaltered blots are available in the Fig. S5.

### Immunofluorescence

Cells seeded on coverslips in 24-well plates were fixed with 4% paraformaldehyde for 10 min at 37°C followed by quenching with the addition of 0.1 M glycine in PBS for 10 min at RT. Fixed cells were permeabilized with 0.1% Triton X-100 in PBS for 5 min and then blocked with goat serum [0.1% (v/v) diluted in PBS] and BSA (5 mg/ml in PBS). Primary antibodies diluted in PBS were added overnight at 4°C. Cells were washed three times with PBS and secondary antibodies (diluted 1:500 in PBS) were added for 1 h at RT. After washing three times with PBS, coverslips were mounted with Mowiol.

For staining of mitochondria, Mitotracker Orange CMXRos (75 nM, M7510, Thermo Fisher Scientific) was added prior to fixation of the cells. The stain was diluted using DMEM and added to the cells for 15 min at 37°C, 5% CO_2_. Cells were washed once with PBS and then fixed for further staining. Staining of Halo-tagged Miro proteins was carried out with HaloTag^®^ Oregon Green^®^ ligand (1 µM) by diluting the stain with complete DMEM and adding it to the cells for 15 min at 37°C, 5% CO_2_. Cells were incubated in DMEM for 30 min at 37°C, 5% CO_2_ to wash away unbound ligand. Cells were fixed for further processing after two washes with PBS.

### Preparation of cultured cells for transmission electron microscopy

Cells were seeded on coverslips in 24-well plates for 24–48 h before being fixed. Half of the DMEM cell medium was replaced by 4% paraformaldehyde and 4% glutaraldehyde in cacodylate buffer (100 mM sodium cacodylate pH 7.4, 2 mM CaCl_2_) and incubated for 10 min. The mixture of medium and buffer was removed and replaced by 2% paraformaldehyde and 2% glutaraldehyde in cacodylate buffer for 2 h at RT. Cells were washed three times with cacodylate buffer and post-fixed with 1% osmium tetroxide in cacodylate buffer for 30 min on ice followed by 30 min at RT. Afterwards samples were washed in distilled water and then dehydrated in a graded ethanol series starting with 70% ethanol overnight at 4°C followed by two times with 90% ethanol and 96% ethanol each for 15 min at RT. Finally, samples were incubated thrice for 30 min at RT with 100% anhydrous ethanol. Samples were infiltrated with increasing concentrations of embedding medium freshly prepared by mixing 60% (v/v) Epon I (37.48 g Epon 812 (Serva), 49.50 g dodecenyl succine anhydride (Agar) and 40% (v/v) Epon II (60.45 g Epon 812, 52.98 g methylnadic anhydride) (Serva). Samples were incubated at RT for 1 h in 3:1 anhydrous ethanol and embedding medium, followed by 1:1 and 1:3 anhydrous ethanol and embedding medium, respectively. Afterwards samples were incubated under the hood in 100% embedding medium overnight at RT followed by replacement with embedding medium. Gelatine capsules with removed bottom were placed on the coverslips with attached cells and a thin layer of embedding medium and surface-dried for ∼6 h at 60°C. Subsequently, the capsule was completely filled with embedding medium and polymerized at 60°C for 72 h. The coverslip was carefully removed by alternate incubation of the polymerized resin in liquid nitrogen and hot water. The Epon blocks were trimmed and ultrathin (50 nm) sections were cut using an ultramicrotome (Ultracut E, Reichert-Jung). Sections were mounted on 75 mesh formvar-coated copper grids (PLANO) and contrasted with 1% uranyl acetate for 30 min. Sections were analyzed at 100 kV with a Zeiss Libra 120 transmission electron microscope.

### Myo19–FLAG and Mtx3–FLAG affinity purifications

Cells stably expressing either FLAG-tagged Myo19 or Mtx3 were seeded in 15-cm cell culture dishes and treated overnight with 10 mM sodium butyrate to enhance expression of the recombinant protein. The next day, cells were washed with cold PBS and harvested by scraping. The cell pellet obtained was washed once more with cold PBS and then resuspended in 1 ml lysis buffer (150 mM NaCl, 20 mM HEPES pH 7.4, 2 mM MgCl_2_, 1 mM EGTA, 0.5% Triton X-100 and 2 mM ATP), which was supplemented with 0.1 mg/ml Pefabloc, 0.01 mg/ml leupeptin and 0.02 U/ml aprotinin. The lysate was kept on ice for 30 min and then centrifuged at 13,400 ***g*** for 30 min at 4°C. Meanwhile, ∼200 µl (packed volume) anti-FLAG agarose beads were equilibrated with 5 ml lysis buffer (without detergent) by washing and spinning at 1000 ***g*** for 5 min at 4°C. The supernatant of the lysate was added to the beads and the tube was kept on a tube roller for 2 h at 4°C. Next, the agarose beads were washed once with 1 ml lysis buffer and twice with 1 ml wash buffer (50 mM KCl, 10 mM HEPES pH 7.4, 2 mM MgCl_2_, 1 mM EGTA and 1 mM β-mercaptoethanol). For elution of proteins, 25 µl of 5 mg/ml Flag peptide was diluted with 475 µl wash buffer and 100 µl aliquots of the elution buffer were added to the beads and incubated for 5 min on ice. The incubation and elution step were repeated four times with Flag peptide.

Halo-Trap was carried out according to the protocol provided by the manufacturer using ChromoTek Halo-Trap Agarose beads (Proteintech, Munich, Germany).

### Lipidomic analysis of isolated mitochondria

Mitochondria were isolated with Qproteome^TM^ Mitochondria Isolation Kit (Qiagen) accordingly to the manufacturers' protocol. Briefly, cells were seeded at density 5×10^6^ in 15 cm plate and grown overnight at 37°C, 95% humidity and 5% CO_2_. Cells were washed with ice-cold 1× PBS by centrifugation (500 ***g***, 10 min at 4°C), resuspended in ice-cold lysis buffer (Qiagen) and incubated for 10 min at 4°C. Lysates were centrifuged at 1000 ***g*** for 10 min at 4°C to isolate cytosolic proteins. Cell pellets were disrupted in 1.5 ml Disruption buffer (Qiagen) using Potter-Elvehjem Tissue Homogeniser and further centrifuged at 1000 ***g*** for 10 min at 4°C to pellet nuclei and cell debris. Supernatants were transferred to a clean 1.5 ml tube and centrifuged at 6000 ***g*** for 10 min at 4°C to pellet mitochondria. To obtain high-purity mitochondria, pellets were resuspended in mitochondria purification buffer (Qiagen) and centrifuged in a mitochondria purification–disruption buffer gradient at 14,000 ***g*** for 15 min at 4°C. The mitochondria band was diluted in mitochondria storage buffer (Qiagen) and centrifuged at 8000 ***g*** for 10 min at 4°C. This step was repeated several times until mitochondria formed a pellet at the bottom of the tube. Mitochondria were stored in mitochondria storage buffer (Qiagen) at −80°C following flash freezing in liquid nitrogen. Protein concentrations of the mitochondrial fractions isolated from WT, Miro DKO and Myo19 KO cells were determined by BCA assay. 20 µg of mitochondria from each sample were aliquoted and flash frozen in liquid Nitrogen. The samples were then subjected to mass-spectrometric lipid analysis. Two types of techniques were employed to estimate lipids in mitochondrial samples: (1) Fourier transform mass spectrometry on a hybrid quadrupole-Orbitrap mass spectrometer (high-mass resolution); (2) tandem mass spectrometry on a triple quadrupole mass spectrometer (low-mass resolution). Percentage levels of each lipid were calculated for all of the samples.

For quantitative lipidomics, internal standards were added prior to lipid extraction. An amount of 20 µg protein was subjected to lipid extraction according to the protocol by (Bligh and Dyer, 1959). The analysis of lipids was performed by direct flow injection analysis (FIA) using a triple quadrupole mass spectrometer (FIA-MS/MS) and a high-resolution hybrid quadrupole-Orbitrap mass spectrometer (FIA-FTMS). FIA-MS/MS was performed in positive ion mode using the analytical setup and strategy described previously (Liebisch et al., 2004). A fragment ion of *m/z* 184 was used for lysophosphatidylcholines (LPC) (Liebisch et al., 2002). The following neutral losses were applied: phosphatidylethanolamine (PE) and lysophosphatidylethanolamine (LPE) 141, phosphatidylserine (PS) 185, phosphatidylglycerol (PG) 189 and phosphatidylinositol (PI) 277 (Matyash et al., 2008). Sphingosine based ceramides (Cer) and hexosylceramides (Hex-Cer) were analyzed using a fragment ion of *m/z* 264 (Liebisch et al., 1999). PE-based plasmalogens (PE P) were analyzed according to the principles described by Zemski-Berry (Zemski Berry and Murphy, 2004). Cardiolipin was monitored by diglycerol fragment ions (Scherer et al., 2010). Glycerophospholipid species annotation was based on the assumption of even numbered carbon chains only.

A detailed description of the FIA-FTMS method was published recently (Höring et al., 2021). Triglycerides (TGs), diglycerides (DGs) and cholesterol esters (CEs) were recorded in positive ion mode at *m*/*z* 500–1000 as [M+NH_4_]^+^ at a target resolution of 140,000 (at 200 *m*/*z*). CE species were corrected for their species-specific response (Höring et al., 2019). Phosphatidylcholines (PCs), PC ether (PC O) and sphingomyelins (SMs) were analyzed in negative ion mode at *m*/*z* 520–960 as [M+HCOO]^−^ at the same resolution setting. Analysis of free cholesterol (FC) was performed by multiplexed acquisition (MSX) of the [M+NH_4_]^+^ of FC and the deuterated internal standard (FC[D7]) (Höring et al., 2019). Free fatty acids (FFAs) were analyzed in negative ion mode at *m*/*z* 150–450 as [M–H]^−^ dissolved in methanol/chloroform=5/1 (v/v) containing 0.005% dimethylamine.

### Mitochondrial stress test – measurement of OCR

To measure mitochondrial function in cells, the XF Cell Mito Stress Test (Seahorse Bioscience) was used to determine OCRs. The key parameters of mitochondrial function were assessed by injections of respiration modulators, which target components of the electron transport chain. First the sensor cartridge was calibrated overnight with 200 µl of XF Calibrant per well at 37°C. The next day, 10^5^ cells were seeded in a 96-well XF Cell Culture Microplate in complete DMEM and incubated for 4 h at 37°C, 5% CO_2_ and 95% humidity, until all cells were attached. Afterwards, cell medium was replaced with 100 µl of XF Base medium supplemented with 4.5 g/l glucose, 1 mM pyruvate and 2 mM glutamine. Cells were incubated in assay medium for 1 h at 37°C, 5% CO_2_. In the meantime, the sensor cartridge was filled with 1 µM oligomycin in port A, 0.5 µM FCCP in port B and 0.5 µM rotenone and antimycin A (along with Hoechst 33342 to label cell nuclei) in port C and a calibration of the plate with the loaded sensor cartridge was performed. Following calibration and equilibration of the cell culture microplate, the assay was run with the XF Extracellular Flux Analyzer. All parameters were automatically generated and calculated by the XF Mito Stress Test Report Generator (Seahorse Bioscience). After the OCR measurements, cell number in each well was calculated by Cytation and imported into the Wave program for normalization of OCR measurements with the cell number.

### Image acquisition and analysis

Fluorescence images were acquired using confocal laser scanning microscope Zeiss LSM800 or Leica TCS SP8 SMD. Ultrastructural images were acquired using a Zeiss Libra120 transmission electron microscope. Confocal and transmission electron microscopy (TEM) images were analyzed by ImageJ software (NIH).

Mitochondria from WT, Miro DKO and rescues of Miro DKO cells were classified into three categories based on the cristae density: (1) normal cristae (WT-like); (2) intermediate (>50% of the area devoid of cristae); and (3) aberrant (severe loss of cristae). This was undertaken for 120–150 mitochondria per cell type by a researcher who was unaware of the experimental conditions. The percentage of mitochondria in each category in a given cell type was calculated and plotted. To quantify mitochondrial Myo19 fluorescence intensity, mitochondrial staining of Mitotracker Orange was used to create a mask. The mean Myo19 fluorescence intensity for the mitochondrial area was determined and mean background intensity was subtracted from this value. Mitochondrial cristae width in WT, Mtx3 KO and Mtx3KO-Mtx3Halo cells was calculated using ImageJ. The distance between a membrane fold forming a crista (perpendicular to the length of crista) was measured and the mean width was calculated for all cristae analyzed per mitochondrion. Mitochondria were analyzed for ER membrane contacts (distance between ER and mitochondria membranes less than 35 nm) using TEM images of WT, Mtx3 KO and Mtx3KO-Mtx3Halo cells.

### Statistical analysis

Graphs for OCR were generated by the Wave^®^ program by Agilent as Microsoft Office Excel files. Data were analyzed using OriginPro 2022 (OriginLab Corporation). For pairwise comparisons, a paired two sample *t*-test was employed. One-way ANOVA with indicated post-tests was employed in case of comparison of means of data, as indicated in the figure legends. Bar graphs show mean±s.e.m. values. Significance values are indicated as *P≤0.05, **P≤0.01 and ***P≤0.001.

## Supplementary Material



10.1242/joces.263637_sup1 Supplementary information
