# Supplementary material for: Regulation of mitochondrial cristae organization by Myo19, Miro1 and Miro2, and metaxin 3
Source: J Cell Sci. 2025 Mar 6;138(9):JCS263637. doi: 10.1242/jcs.263637 (PMC11925395; doi:10.1242/jcs.263637)
Supplement: Supplementary information [file joces-138-263637-s1.pdf]

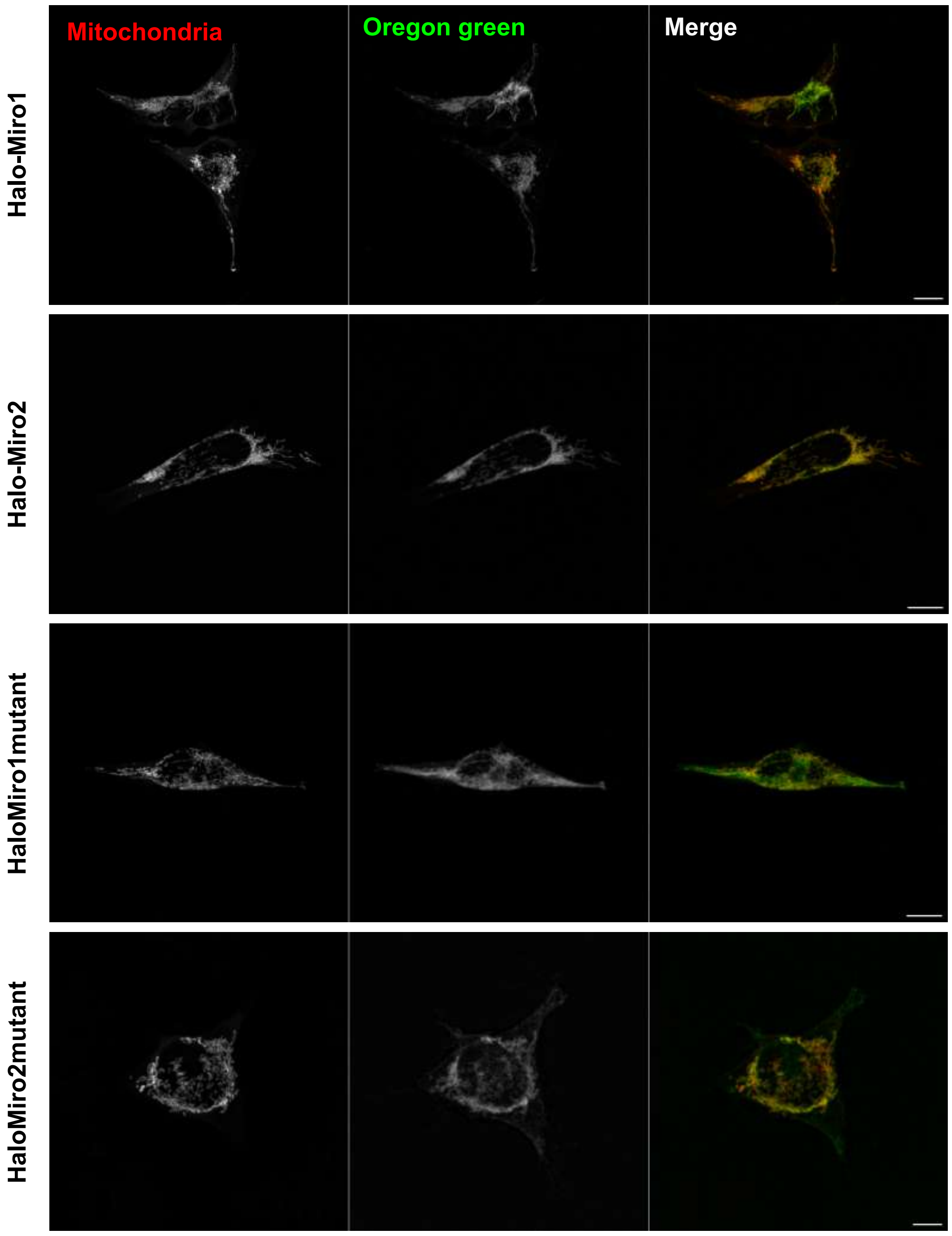

Fig. S1. Confocal images of Miro DKO-cells expressing in (B) Halo-Miro1 and Halo-Miro2 and in (C) Halo-Miro1mutant and Halo-Miro2mutant. The Halo-Tag was labelled with Oregon green. Mitochondria were stained with Mitotracker Orange. n= 20 -30 cells per experiment . All scale bars, 10 μm.

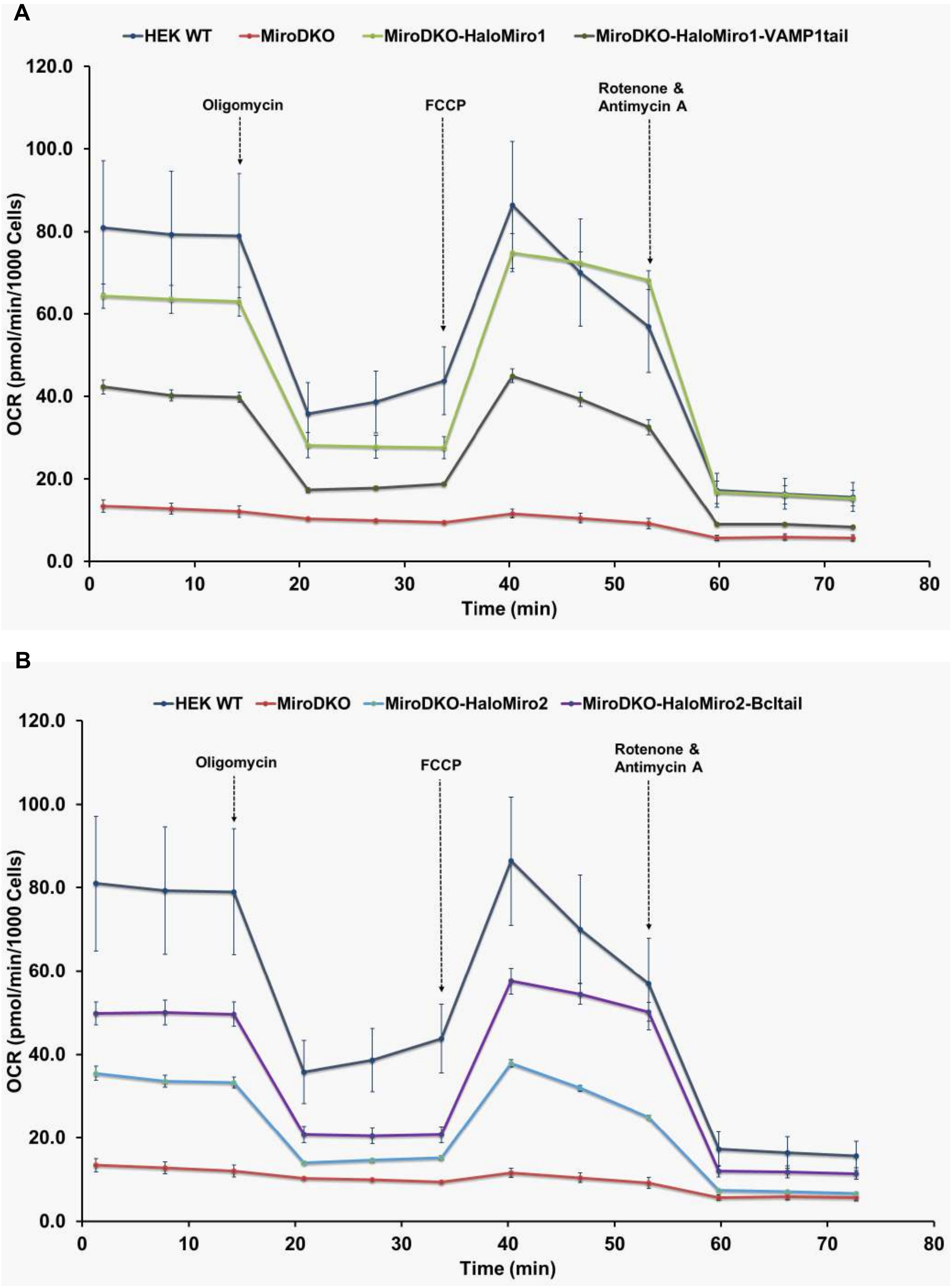

Fig. S2. Oxygen consumption rates of WT, Miro DKO and different Miro DKO rescue cells. (A) Graph of oxygen consumption rate (OCR) profiles of WT, Miro DKO, Miro DKO - HaloMiro1 and Miro DKO -HaloMiro1-VAMP1tail (Miro DKO-HaloMiro1mutant) (B) OCR profiles of WT, Miro DKO, Miro DKO -HaloMiro2 and Miro DKO-haloMiro2Bcl-xLtail (Miro DKO -HaloMiro2mutant). All of the graphs are from one representative experiment (3 wells for each cell type).

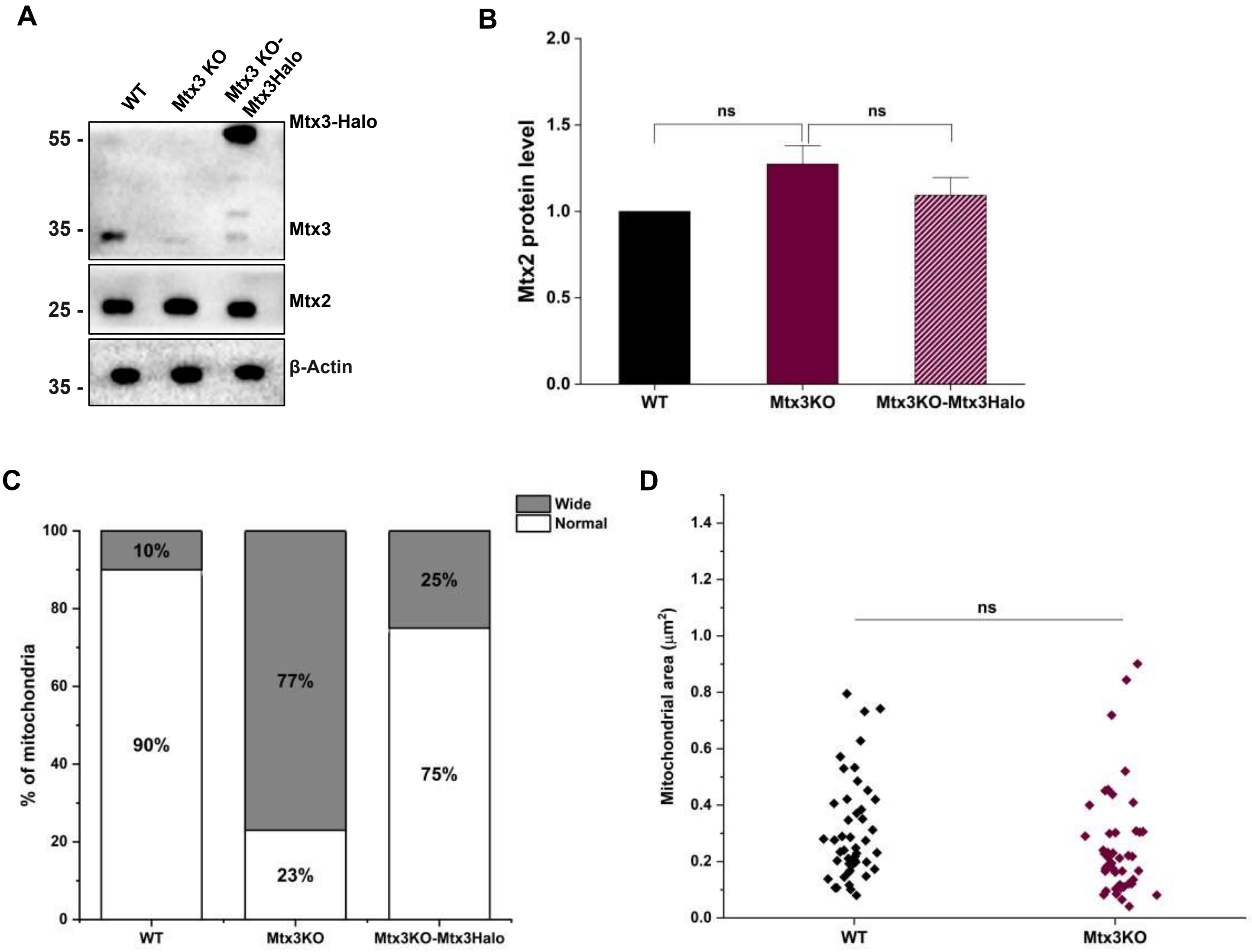

Fig. S3. Analysis of Mtx3 KO cells. (A) Western blot analysis of Mtx3, Mtx2 and β-actin in WT, Mtx3 KO and Mtx3 KO-Mtx3Halo cell lysates . β-actin served as loading control. (B) Quantification of Mtx2 in the indicated samples. Two-sample t test was used for calculating statistical significance. (C) Comparative analysis of mitochondria with normal and wide cristae for the indicated cell types. 150-200 mitochondria were analyzed per cell type. (D) Analysis of m itochondrial area in WT and Mtx3 KO cells using transmission electron microscopy. Statistical analysis with one -way ANOVA and Tukey-Kramer post-hoc test; 40- 50 mitochondria analyzed per cell type.

Journal of Cell Science • Supplementary information

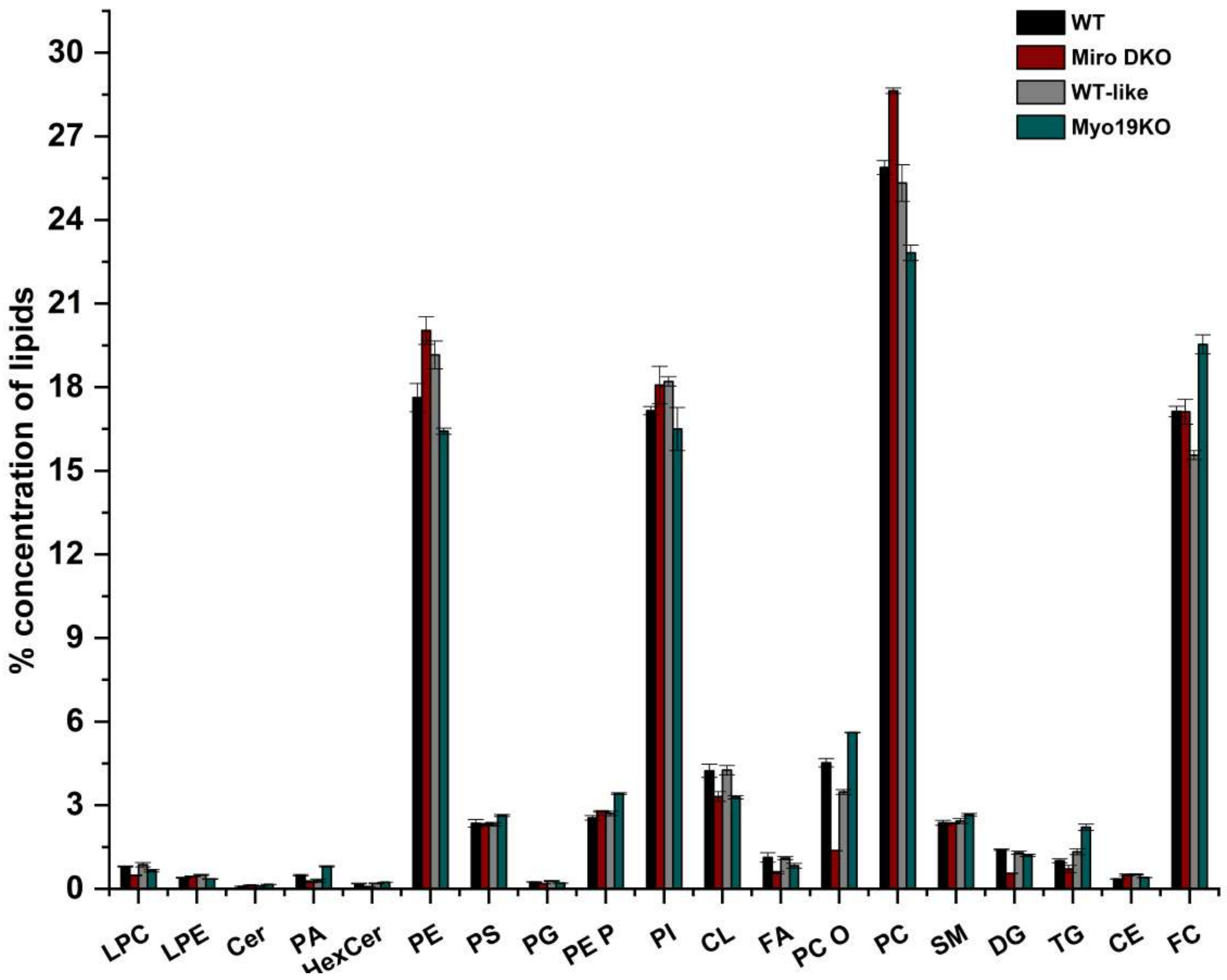

Fig. S4. Lipidomic analysis of WT, Miro DKO and Myo19 KO mitochondria. Percentage levels of indicated lipids from WT and Miro DKO mitochondria plotted as bar graph (mean+SE from 3 independent experiments).

Journal of Cell Science • Supplementary information

Fig.1A

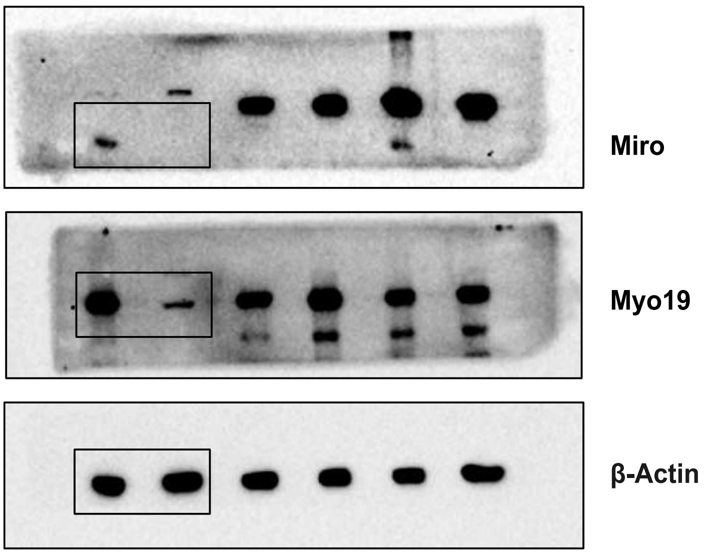

Fig.2B

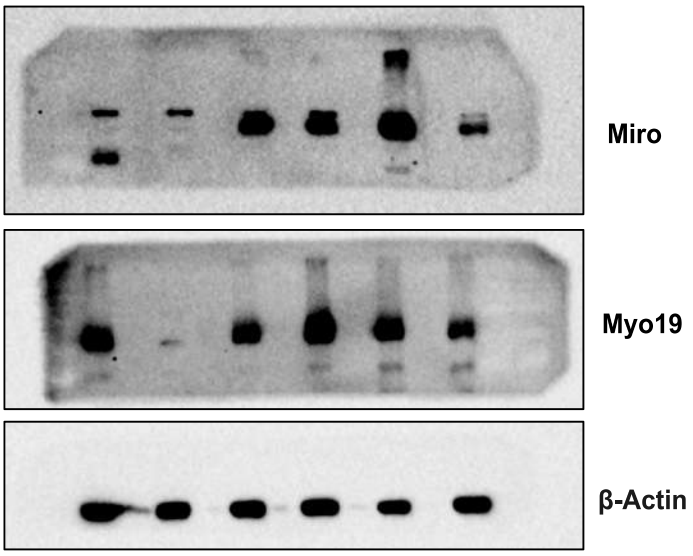

Fig.2F

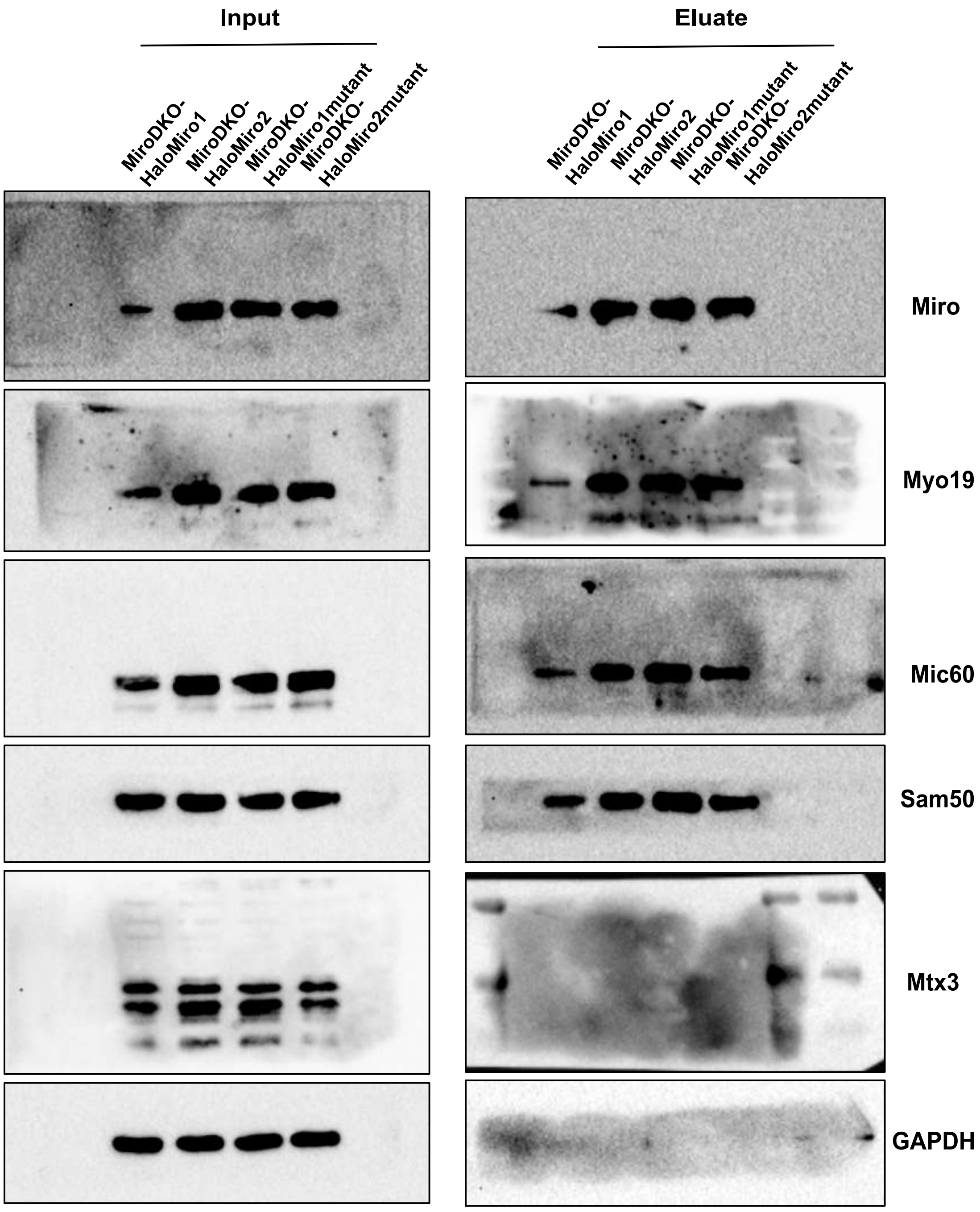

Journal of Cell Science • Supplementary information

Fig.4B

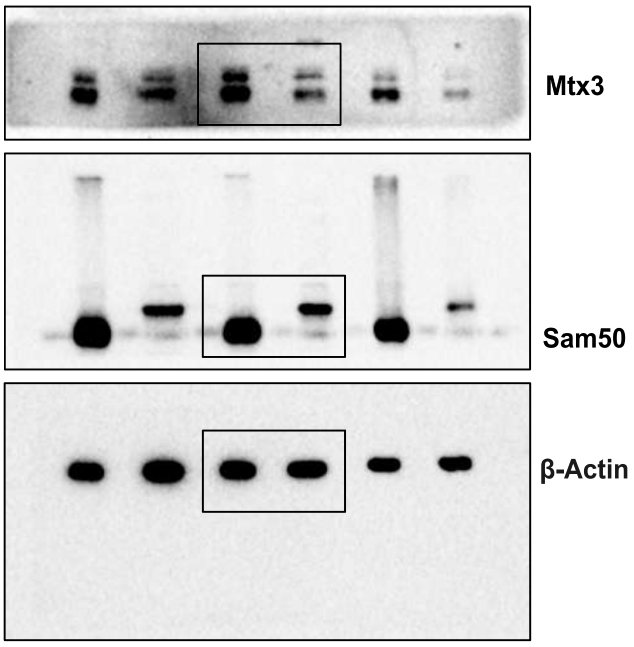

Fig.4C

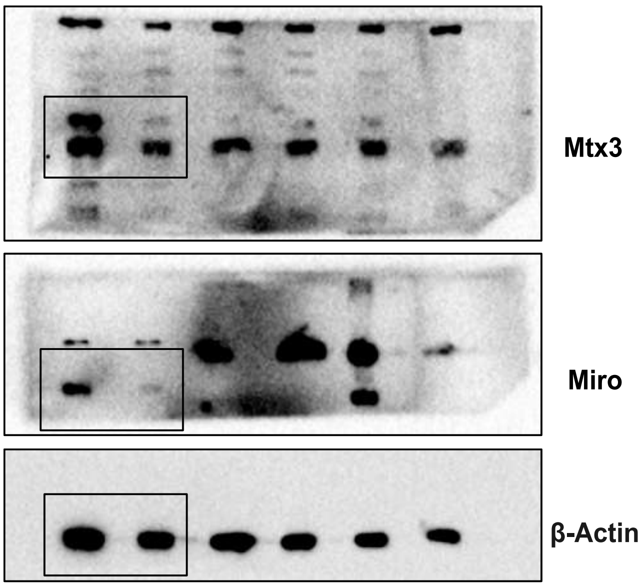

Fig.5A

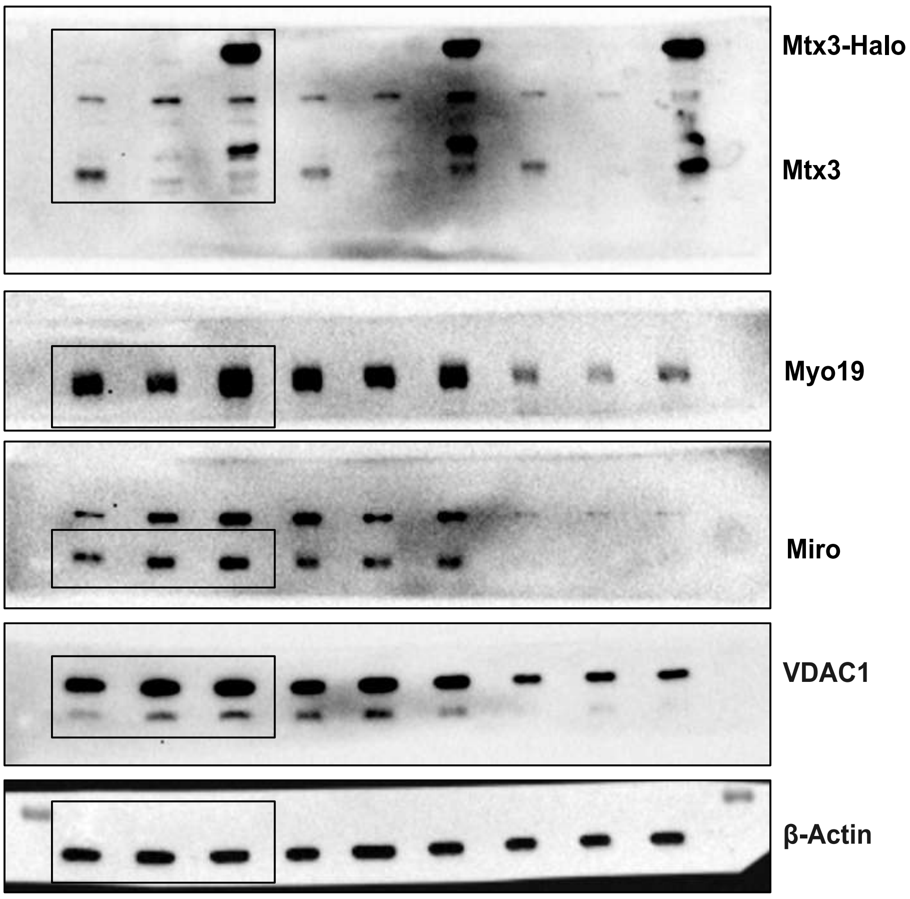

Journal of Cell Science • Supplementary information

Fig.7

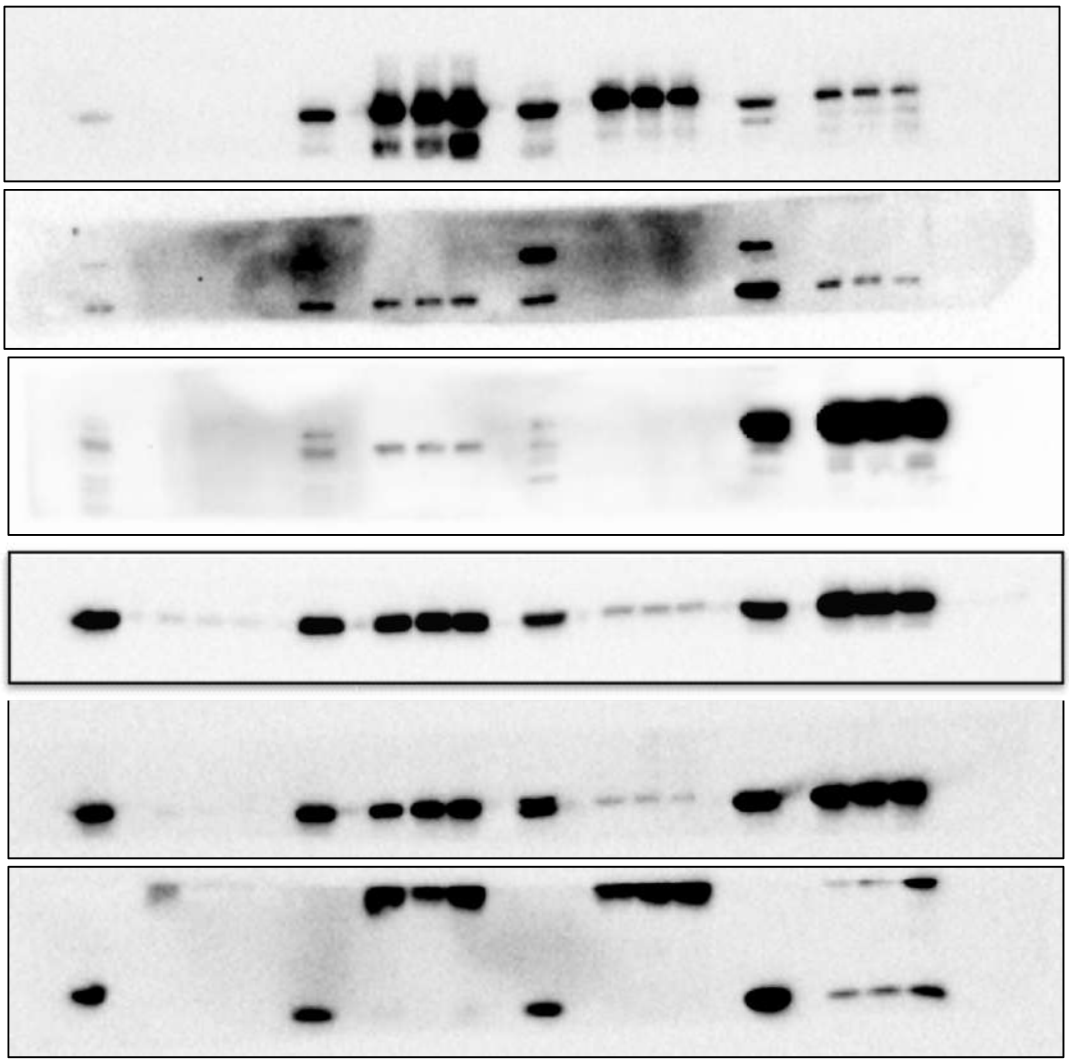

Fig.S3A

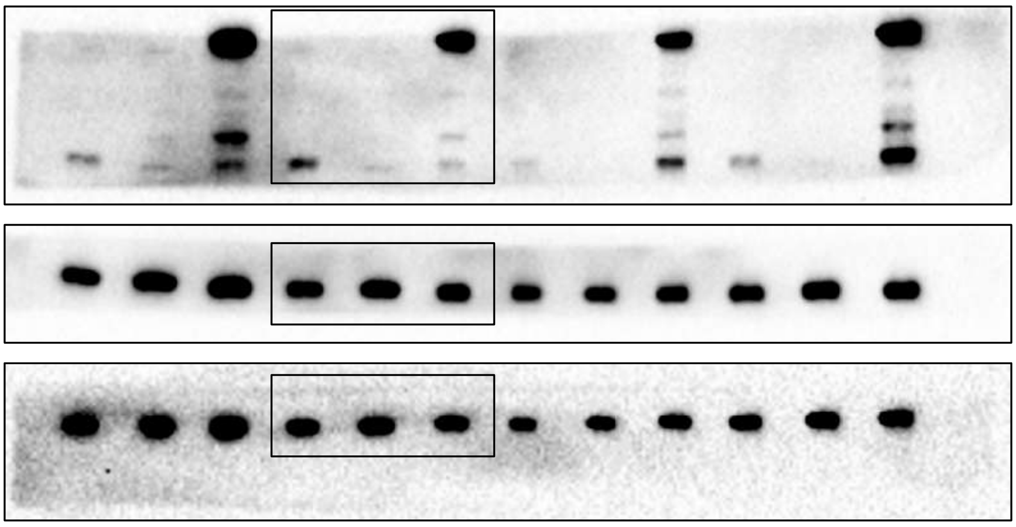

Fig. S5. Blot Transparency.
