# Supplementary material for: Regulation of mitochondrial cristae organization by Myo19, Miro1 and Miro2, and metaxin 3
Source: J Cell Sci. 2025 Mar 6;138(9):JCS263637. doi: 10.1242/jcs.263637 (PMC11925395; doi:10.1242/jcs.263637)
Supplement: Supplementary file 1 [file joces-138-263637_review_history.pdf]

Journal of Cell Science

Regulation of mitochondrial cristae organization by Myo19, Miro1 and Miro2, and metaxin 3

Samruddhi S. Shembekar, Petra Nikolaus, Ulrike Honnert, Marcus Höring, Aya Attia, Karin Topp, Birgit Lohmann, Gerhard Liebisch and Martin Baehler
DOI: 10.1242/jcs.263637

Editor: Jennifer Lippincott-Schwartz

Review timeline

Original submission:	29 February 2024
Editorial decision:	15 April 2024
First revision received:	22 November 2024
Accepted:	13 January 2025

Original submission

First decision letter

MS ID#: JOCES/2024/262073

MS TITLE: The association of Myo19 with the MIB/MICOS complex of mitochondria: Myo19 is linked to SAMM50 and Mic60 independently of Miro1/2 and Metaxin 3

AUTHORS: Samruddhi S Shembekar, Petra Nikolaus, Ulrike Honnert, Aya Attia, Karin Topp, Birgit Lohmann, and Martin Baehler
ARTICLE TYPE: Research Article

We have now reached a decision on the above manuscript.

As you will see, the reviewers raise a number of substantial criticisms that prevent me from accepting the paper at this stage. They suggest, however, that a revised version might prove acceptable, if you can address their concerns. If you think that you can deal satisfactorily with the criticisms on revision, I would be pleased to see a revised manuscript. We would then return it to the reviewers.

Reviewer 1 Advance Summary and Potential Significance to Field:

The manuscript by Shembekar et al. studies the link between MYO19 and the MIB/MICOS complex focusing specifically on Miro1/2 and the SAM complex component, Metaxin 3. More specifically, the authors conclude that Metaxin 3 is required for the stability of MYO19 independently of Miro1/2 and that Miro and Metaxin 3 are required to maintain the ultrastructure of the mitochondria. Many experiments are repeats of previously published work, but the study makes interesting new findings, including that metaxin 3-myo19 and miro are not part of the same complex, and the fact that Myo19 needs Metaxin to interact with Miro (despite not being part of the same complex).The results are generally convincing and well controlled.

Reviewer 1 Comments for the Author:
Major points to consider:
1.Myo19 localization to mitochondria is blunted but not entirely abrogated by Miro1/2 KO, something that was observed before (e.g. PMID 29311115). Metaxin3 could be a great candidate for the missing miro-independent Myo19-recruiting activity on mitochondria (cf. PMID 31479585). It is a

© 2025. Published by The Company of Biologists under the terms of the Creative Commons Attribution License (https://creativecommons.org/licenses/by/4.0/).

pity that the manuscript does not go as far as to test this hypothesis. This could be done rather easily by any number of ways. For instance, RNAi of Metaxin3 in miro1/2 KO cells should cause the loss of the remaining mitochondrial. A similar result could be obtained by CRISPR KO of metaxin3 instead of RNAi (though likely more work). Alternatively, one could express GFP-tagged point mutants of Myo19 that cannot interact with Miro (cf PMID 38267654). These mutants should still be mitochondrial in WT but not in metaxin 3 KO cell lines. In fact, one could argue that these experiments are required to fully support the title of the study which mentions association of Myo19 to the MIB complex “independently of Miro1/2 and Metaxin 3”. In reality, this manuscript shows association independent of Miro1/2 OR metaxin 3 individually, but not independent of both together.

2.One potentially exciting result is that MYO19 does not interact with Miro1/2 in the absence of Metaxin 3 (Figure 9) with only modest changes in the stability of MYO19 (Figure 7); This finding comes as a surprise given that the direct interaction between Myo19 and Miro is well established by assays, like yeast two hybrid (that do not involve metaxin!). Moreover, there is extensive work showing that MYO19 must interact with Miro1/2 to be stable and mitochondria-localized. How do the authors reconcile this? How can Myo19 be relatively stable in the absence of metaxin3 even though it does not interact with Miro in these conditions? How can it be mostly mitochondrial in metaxin 3 KO while it is mostly cytosolic in miro1/2 KO. One possibility could be that Myo19-Miro interaction is not required ‘in cis’ for Myo19 stability and mitochondrial stability, but that miro protein do something to mitochondria ‘in trans’, which helps stabilizing Myo19, perhaps change its lipidome via VPS13D, or its proteome via Parkin? The other possibility could be that the Myo19-Miro interaction is only partially dependent on metaxin3, and therefore, metaxin 3 KO causes only a reduction of binding and therefore a milder phenotype than Miro/2 KO. Regardless, these points should be discussed thoroughly in the discussion, as this bit of data is one of the truly new finding of this study.

3.Two of the big findings are based on negative results (namely, 1) Miro IP pulls-down Myo19 but does not pull down Metaxin 3, and 2) MYO19 does not interact with Miro in Metaxin 3 knockout cells). Negative results should be very well controlled with positive controls, meaning including wild type cells alongside the Metaxin 3 knockout pulldown experiment in Figure 9 and running everything on the same blot. Comparing Myo19 IP to Metaxin3 IP for Miro pull down on the same blots.

4.A large number of main figures are repeats of previously published work (e.g. Fig. 1 and 2). The main issue with this is that the novel results are somewhat lost amongst those that are already known and the new conclusions of the paper are a little tricky to follow. Large amounts of text are given to describing and discussing previous data leaving little room for new work, (see point 2).

Minor points:
Fig 3A does not show the halo tags.

-p 5 “a significant part of the Myo19 signal was cytosolic in Miro DKO cells, although an association with mitochondria was still discernible (Fig. 2 D).” Although this result appears sound (and consistent with previous literature) it seems difficult to follow the authors in their conclusion by the inspection of Fig 2D alone.

-p 14 “One of the possible reasons for differences in ERMCS numbers could be corresponding differences in surface area of the mitochondria. However, we found no significant difference in the area between WT and Mtx3 KO mitochondria (Fig. S2 D).”. To be complete, differences in ERMCS numbers could also be due to corresponding differences in the surface area of the ER.

Reviewer 2 Advance Summary and Potential Significance to Field:

Shembekar et al performed various cellular IP and ICC in genetically edited cells to elucidate the physical relationship among Myo19, Miro, and Metaxin3. Most of the experiments are properly done and controls are included. A major concern is that the conclusion is based on cellular IP with overexpressed proteins (Fig. 1) or in KO cells with expressed proteins. Overexpression could change the native stoichiometry of the endogenous protein complex leading to false positive/negative

© 2025. Published by The Company of Biologists under the terms of the Creative Commons Attribution License (https://creativecommons.org/licenses/by/4.0/).

interaction. In addition, cellular IP cannot determine a direct interaction since proteins could indirectly bind to other proteins in the same complex. An in vitro system is essential to confirm how purified Miro, Myo19, Metaxin3 proteins bind to each other. This system will also solve the common protein stability issue when one of the components in the complex is missing.

Reviewer 2 Comments for the Author:
The conclusion of the C-tail of Miro being not essential for the interaction: the authors swapped the C-tail of Miro1 with other OMM proteins, rather than deleting it. It is possible the swapped new sequence still allows Miro1 to interact with MICOS via other proteins in the OMM and IMS. Li et al 2021 (Nature Metabolism) reported deleting the last C-terminal 11 amino acids protruding into the IMS of fly Miro compromises its interaction with fly MIC60. The authors could delete the small fragment of Miro1/2 in the IMS while keeping the OMM domain and detect their interaction with MICOS. In addition, the in vitro system would be very useful to verify the conclusion whether the cytosolic fraction of Miro1 interacts with MICOS/Myo19/Metaxins by only purifying the N-terminal cytosolic portion of Miro1 and perform IP in vitro.

Miro DKO cells should be used in Fig. 9 to confirm MICOS binds to Myo19 independent of Miro.

other:
1. Multiple places: please cite the first paper that discovered Miro1 and MICOS (MIC60) interaction: Tsai et al 2017, MBoC

-Introduction:
“Miro proteins have been reported to form a complex with MICOS (Mitochondrial contact site and cristae organization system) proteins to maintain the structural organization of cristae (Modi et al., 2019)”

-Results Section 2:
“The outer mitochondrial membrane receptors Miro1/2 that were co-precipitated with Myo19, have been reported to be associated with the MICOS complex (Modi et al., 2019).”

2. Fig. 2D, 4C, 6E, 7D ICC images are too dark/faint to see any details. The authors could also consider zooming in on details by enlarging a small area inside the cell.

First revision

Author response to reviewers' comments

Reviewer 1 Advance Summary and Potential Significance to Field:
The manuscript by Shembekar et al. studies the link between MYO19 and the MIB/MICOS complex focusing specifically on Miro1/2 and the SAM complex component, Metaxin 3. More specifically, the authors conclude that Metaxin 3 is required for the stability of MYO19 independently of Miro1/2 and that Miro and Metaxin 3 are required to maintain the ultrastructure of the mitochondria. Many experiments are repeats of previously published work, but the study makes interesting new findings, including that metaxin 3-myo19 and miro are not part of the same complex, and the fact that Myo19 needs Metaxin to interact with Miro (despite not being part of the same complex).The results are generally convincing and well controlled.

We have revised the manuscript to emphasize more the new findings, reduced the repeat findings (among others deleted Fig.1) and added new lipid data of purified mitochondria from Miro DKO and Myo19 KO cells.

Reviewer 1 Comments for the Author:
Major points to consider:
1.Myo19 localization to mitochondria is blunted but not entirely abrogated by Miro1/2 KO,

© 2025. Published by The Company of Biologists under the terms of the Creative Commons Attribution License (https://creativecommons.org/licenses/by/4.0/).

something that was observed before (e.g. PMID 29311115). Metaxin3 could be a great candidate for the missing miro-independent Myo19-recruiting activity on mitochondria (cf. PMID 31479585). It is a pity that the manuscript does not go as far as to test this hypothesis. This could be done rather easily by any number of ways. For instance, RNAi of Metaxin3 in miro1/2 KO cells should cause the loss of the remaining mitochondrial. A similar result could be obtained by CRISPR KO of metaxin3 instead of RNAi (though likely more work). Alternatively, one could express GFP-tagged point mutants of Myo19 that cannot interact with Miro (cf PMID 38267654). These mutants should still be mitochondrial in WT but not in metaxin 3 KO cell lines. In fact, one could argue that these experiments are required to fully support the title of the study which mentions association of Myo19 to the MIB complex "independently of Miro1/2 and Metaxin 3". In reality, this manuscript shows association independent of Miro1/2 OR metaxin 3 individually, but not independent of both together.

We thank the reviewer for these suggestions. A serious limitation in the experimental design is the fact, that Myo19 is downregulated concomitantly with the loss of Miro1/2. Miro stabilizes Myo19 and therefore, it is predicted that in stably transfected cells used in our current study, the Myo19 mutant that cannot interact with Miro will not be stable. A notion that was supported by transient transfections. This seriously compromises comparisons with affinity purifications from wildtype cells. However, a re-examination of affinity purifications of Myo19 from metaxin-3 KO cells using all FLAG-tagged instead of also Halo-tagged constructs revealed that MIB components are strongly reduced in comparison to wildtype cells and that metaxin-3 is also connected to Miro1/2 when it is affinity purified with FLAG- tag (in contrast to the Halo-pulldowns used previously). It, therefore, appears that metaxin-3 plays a significant role in linking Miro and Myo19 to the MIB complex. These new results together with the newly included lipid data led us to modify the title of the manuscript.

2. One potentially exciting result is that MYO19 does not interact with Miro1/2 in the absence of Metaxin 3 (Figure 9) with only modest changes in the stability of MYO19 (Figure 7); This finding comes as a surprise given that the direct interaction between Myo19 and Miro is well established by assays, like yeast two hybrid (that do not involve metaxin!). Moreover, there is extensive work showing that MYO19 must interact with Miro1/2 to be stable and mitochondria-localized. How do the authors reconcile this? How can Myo19 be relatively stable in the absence of metaxin3 even though it does not interact with Miro in these conditions? How can it be mostly mitochondrial in metaxin 3 KO while it is mostly cytosolic in miro1/2 KO. One possibility could be that Myo19-Miro interaction is not required 'in cis' for Myo19 stability and mitochondrial stability, but that miro protein do something to mitochondria 'in trans', which helps stabilizing Myo19, perhaps change its lipidome via VPS13D, or its proteome via Parkin? The other possibility could be that the Myo19-Miro interaction is only partially dependent on metaxin3, and therefore, metaxin 3 KO causes only a reduction of binding and therefore a milder phenotype than Miro/2 KO. Regardless, these points should be discussed thoroughly in the discussion, as this bit of data is one of the truly new finding of this study.

The reviewer points out correctly that Myo19 can interact directly with Miro1/2, as previously shown e.g. in *in vitro* biochemical experiments or *in vivo* by compeeeon with TRAK binding (Oeding et al., 2018). This is also supported by the Halo pulldowns of Halo-tagged Miro constructs shown here. Our new results performed consistently with FLAG-tagged constructs reveal now contrary to our inieal findings that metaxin-3 stabilizes the interaction of Myo19 with Miro1/2 and that metaxin-3 is connecting Myo19 and Miro1/2 to the MIB complex. Furthermore, we report that the linkage of Myo19 to the MIB complex via metaxin-3 is not esseneal for cristae morphology, but probably rather Myo19's involvement in ER-mitochondria contacts and associated mitochondrial lipid composieon as indicated by newly included data (new Fig. 8).

3.Two of the big findings are based on negaeve results (namely, 1) Miro IP pulls-down Myo19 but does not pull down Metaxin 3, and 2) MYO19 does not interact with Miro in Metaxin 3 knockout cells). Negaeve results should be very well controlled with posieve controls, meaning including wild type cells alongside the Metaxin 3 knockout pulldown experiment in

© 2025. Published by The Company of Biologists under the terms of the Creative Commons Attribution License (https://creativecommons.org/licenses/by/4.0/).

Figure 9 and running everything on the same blot. Comparing Myo19 IP to Metaxin3 IP for Miro pull down on the same blots.

We thank the reviewer for requeseng to run the different affinity purifications side by side on the same blot for direct comparison. This is now implemented in the new Figure 7.

4.A large number of main figures are repeats of previously published work (e.g. Fig. 1 and 2). The main issue with this is that the novel results are somewhat lost amongst those that are already known and the new conclusions of the paper are a little tricky to follow. Large amounts of text are given to describing and discussing previous data leaving little room for new work, (see point 2).

To stress more the new conclusions derived from the present work, we have removed Fig. 1, added more new data and modified the text accordingly.

Minor points
-Fig 3A does not show the halo tags.

Fig. 3A (new Fig. 2A) was modified to highlight directly the actual sequence changes introduced to the transmembrane anchors of Miro1 and Miro2. This also resolves the irritations concerning the actual residues present in the intermembrane space.

-p 5 "a significant part of the Myo19 signal was cytosolic in Miro DKO cells, although an association with mitochondria was sell discernible (Fig. 2 D)." Although this result appears sound (and consistent with previous literature) it seems difficult to follow the authors in their conclusion by the inspection of Fig 2D alone.

We have increased the enlargement of the cells a bit (new Fig. 1D), to better visualize this finding.

-p 14 "One of the possible reasons for differences in ERMCS numbers could be corresponding differences in surface area of the mitochondria. However, we found no significant difference in the area between WT and Mtx3 KO mitochondria (Fig. S2 D).". To be complete, differences in ERMCS numbers could also be due to corresponding differences in the surface area of the ER.

We have added this theoreecal possibility.

*****

Reviewer 2 Advance Summary and Poteneal Significance to Field:
Shembekar et al performed various cellular IP and ICC in geneecally edited cells to elucidate the physical relationship among Myo19, Miro, and Metaxin3. Most of the experiments are properly done and controls are included. A major concern is that the conclusion is based on cellular IP with overexpressed proteins (Fig. 1) or in KO cells with expressed proteins. Overexpression could change the stoichio of the endogenous protein complex leading to false positive/negative interaction. In addition, cellular IP cannot determine a direct interaction since proteins could indirectly bind to other proteins in the same complex. An in vitro system is esseneal to confirm how purified Miro, Myo19, Metaxin3 proteins bind to each other. This system will also solve the common protein stability issue when one of the components in the complex is missing.

We agree that caueon should be used when interpreeng data obtained using overexpression of proteins. Overexpression might shin equilibria and influence affinity purifications. This is why we used stably transfected cell lines that express moderate levels of recombinant proteins. Furthermore, Myo19 depends for its stability on Miro and to a lower extent on metaxin3. Previous work has demonstrated that Myo19 interacts directly with Miro (Oeding

© 2025. Published by The Company of Biologists under the terms of the Creative Commons Attribution License (https://creativecommons.org/licenses/by/4.0/).

et al., 2018) and metaxin3 (Shi et al., 2022). In a Turbo-ID screen (Attia et al., in prepartion) we found that metaxin-3 is in proximity of Myo19 and vice versa, but metaxin-3 was not in proximity of Miro2. Also, Myo19 and Miro2 were in proximity of each other. These results are perfectly in agreement with our re-examination of the interactions using FLAG-tagged constructs throughout in the revised version. Namely, that Myo19 is linked to the MIB complex to a large extent through metaxin-3.

Reviewer 2 Comments for the Author:
The conclusion of the C-tail of Miro being not essential for the interaction: the authors swapped the C-tail of Miro1 with other OMM proteins, rather than deleting it. It is possible the swapped new sequence sell allows Miro1 to interact with MICOS via other proteins in the OMM and IMS. Li et al 2021 (Nature Metabolism) reported deleting the last C-terminal 11 amino acids protruding into the IMS of fly Miro compromises its interaction with fly MIC60. The authors could delete the small fragment of Miro1/2 in the IMS while keeping the OMM domain and detect their interaction with MICOS.

We would like to emphasize that whereas fly Miro has 11 amino acid residues protruding into the IMS, human Miro1/2 have only 7 residues protruding into the IMS that are not conserved between Miro1, Miro2 and Drosophila Miro (see sequences below and new Fig. 2A). Deletion of the few residues protruding to the IMS (especially the positively charged ones) would likely abolish OMM insertion. Therefore, we swapped the IMS and transmembrane regions of Miro 1 and Miro2 with unrelated C-terminally anchored mitochondrial OMM proteins. These new anchors contained only 2 and 3 residues protruding to the IMS, respectively. We did not detect any alterations of these constructs in the association with the MICOS complex (new Fig. 2F).

```
Drosophila   603 TMAAFPHLRQFGLMTEDPKLWLKAGLGVAAATMLGFIVLKTIS652
                 TMA +PH+ Q L +          WL+A  G      +LGF + K +
Human Miro1 575  TMAMYPHVTQADLKSS--                           618
Miro1-VAMP-1B    KCNKMMIMLGAICAIIVVVIVRRD
Human Miro2 572  TMAAFPHLVHAELHPS—-                           618
Miro2-Bcl-xL     WFLTGMTVAGVVLLGSLFSRK
```

In addition, the in vitro system would be very useful to verify the conclusion whether the cytosolic fraction of Miro1 interacts with MICOS/Myo19/Metaxins by only purifying the N-terminal cytosolic poreon of Miro1 and perform IP in vitro.

We have shown previously that Myo19 interacts with the cytosolic part of Miro by performing direct *in vitro* biochemical binding experiments (Oeding et al., 2018). This was recently confirmed by Covill- Cooke et al. (2024). Myo19 and metaxin3 are exclusively localized at the cytosolic side of the OMM.

Miro DKO cells should be used in Fig. 9 to confirm MICOS binds to Myo19 independent of Miro.

This would be a worthwhile experiment. However, Miro is required for the stabilization of Myo19 (Lopez- Domenech et al., 2018; Oeding et al., 2018; present manuscript). A loss of Miro goes hand in hand with a loss of Myo19 that prevents a meaningful interpretation of the suggested experiment. Expression of cytosolic Miro lacking the transmembrane anchor (delC) in Miro DKO cells or redirecting Miro to the endosomes (2xFYVE) in Miro DKO cells did not stabilize Myo19 and Myo19 remained downregulated as shown in the immunoblot below.

"NOTE: We have removed unpublished data that had been provided for the referees in confidence."

other:
1. Multiple places: please cite the first paper that discovered Miro1 and MICOS (MIC60) interaction: Tsai et al 2017, MBoC

-Introduction:
"Miro proteins have been reported to form a complex with MICOS (Mitochondrial contact site and cristae organization system) proteins to maintain the structural organization of cristae

© 2025. Published by The Company of Biologists under the terms of the Creative Commons Attribution License (https://creativecommons.org/licenses/by/4.0/).

(Modi et al., 2019)"

-Results Section 2:
"The outer mitochondrial membrane receptors Miro1/2 that were co-precipitated with Myo19, have been reported to be associated with the MICOS complex (Modi et al., 2019)."

We thank the reviewer for this suggestion and apologize for the omission of this reference. We have added the reference at the indicated positions.

2. Fig. 2D, 4C, 6E, 7D ICC images are too dark/faint to see any details. The authors could also consider zooming in on details by enlarging a small area inside the cell.
We have tried to optimize them. The original image files should also contribute to a better visibility of the details.

Second decision letter

MS ID#: jcs.263637R1

MS Title: Regulation of mitochondrial cristae organization by Myo19, Miro1/2 and Metaxin 3

Authors: Martin Baehler
Article Type: Research Article

I am happy to inform you that your paper is now acceptable for publication in J. Cell Science. Congratulations.

© 2025. Published by The Company of Biologists under the terms of the Creative Commons Attribution License (https://creativecommons.org/licenses/by/4.0/).
